# Epidemiology of taeniosis/cysticercosis in Europe, a systematic review: eastern Europe

**DOI:** 10.1186/s13071-018-3153-5

**Published:** 2018-10-30

**Authors:** Chiara Trevisan, Smaragda Sotiraki, Minerva Laranjo-González, Veronique Dermauw, Ziqi Wang, Age Kärssin, Aleksandar Cvetkovikj, Andrea S. Winkler, Annette Abraham, Branko Bobić, Brian Lassen, Carmen Michaela Cretu, Cozma Vasile, Dimitris Arvanitis, Gunita Deksne, Ilievski Boro, István Kucsera, Jacek Karamon, Jovana Stefanovska, Břetislav Koudela, Maja Jurhar Pavlova, Marian Varady, Marina Pavlak, Mindaugas Šarkūnas, Miriam Kaminski, Olgica Djurković-Djaković, Pikka Jokelainen, Dagny Stojčević Jan, Veronika Schmidt, Zorica Dakić, Sarah Gabriël, Pierre Dorny, Brecht Devleesschauwer

**Affiliations:** 1 Department of Biomedical Sciences, Institute of Tropical Medicine, Nationalestraat 155, 2000 Antwerp, Belgium; 2 Department of Veterinary and Animal Sciences, Faculty of Health and Medical Sciences, University of Copenhagen, Dyrlægevej, 100 Frederiksberg, Denmark; 3 Veterinary Research Institute, HAO-DEMETER, Campus Thermi, 57001 Thessaloniki, Greece; 4 IRTA, Centre de Recerca en Sanitat Animal (CReSA, IRTA-UAB), Campus de la Universitat Autònoma de Barcelona, 08193 Bellaterra, Spain; 5 University of Florida College of Medicine, Gainesville, Florida, USA; 6 Veterinary and Food laboratory, Kreutzwaldi 30, 51006 Tartu, Estonia; 7 Institute of Veterinary Medicine and Animal Sciences, Estonian University of Life Sciences, Kreutzwaldi 1, 51006 Tartu, Estonia; 8 Department of Parasitology and Parasitic Diseases, Faculty of Veterinary Medicine, Ss. Cyril and Methodius University in Skopje, Lazar Pop Trajkov 5–7, 1000 Skopje, Former Yugoslav Republic of Macedonia; 9 Centre for Global Health, Department of Neurology, Technical University Munich, Ismaninger Strasse 22, 81675 Munich, Germany; 10 Centre for Global Health, Department of Community Medicine and Global Health, Institute of Health and Society, University of Oslo, Kirkeveien 166, 0450 Oslo, Norway; 11 Centre of Excellence for Food- and Vector-borne Zoonoses, Institute for Medical Research, University of Belgrade, Belgrade, Serbia; 12 Department of Parasitology, Carol Davila University of Medicine and Pharmacy, Colentina Clinical Hospital, Bucharest, Romania; 13 Department of Parasitology, University of Agricultural Sciences and Veterinary Medicine Cluj-Napoca, Cluj-Napoca, Romania; 14 Department of Microbiology, 424 Military General Hospital, Thessaloniki, Greece; 15 Institute of Food Safety, Health and Environment, Riga, Latvia; 16 Faculty of Biology, University of Latvia, Riga, Latvia; 17 Institute for Pathology, Medical Faculty, University “Ss. Cyril and Methodius”, Skopje, Former Yugoslav Republic of Macedonia; 18 Department of Parasitology, National Institute for Public Health, Budapest, Hungary; 19 Department of Parasitology and Invasive Diseases, National Veterinary Research Institute in Pulawy, Pulawy, Poland; 20 Department of Pathology and Parasitology, Faculty of Veterinary Medicine, University of Veterinary and Pharmaceutical Sciences Brno, Palackého tř. 1946/1, 61242 Brno, Czech Republic; 21 Central European Institute of Technology, University of Veterinary and Pharmaceutical Sciences Brno, Palackého tř. 1946/1, 61242 Brno, Czech Republic; 22 Institute for Microbiology and Parasitology, Medical faculty, University “Ss. Cyril and Methodius”, Skopje, Former Yugoslav Republic of Macedonia; 23 Institute of Parasitology, Slovak Academy of Sciences, Košice, Slovakia; 24 Department of Veterinary Economics and Epidemiology, Faculty of Veterinary Medicine, University of Zagreb, Zagreb, Croatia; 25 Lithuanian University of Health Sciences, Kaunas, Lithuania; 26 Department of Neurology, Klinikum rechts der Isar, Technical University Munich, Ismaninger Straße 22, 81675 Munich, Germany; 27 Laboratory of Parasitology, Department of Bacteria, Parasites and Fungi, Infectious Disease Preparedness, Statens Serum Institute, Copenhagen, Denmark; 28 Faculty of Veterinary Medicine, University of Helsinki, Helsinki, Finland; 29 Department of Parasitology and Parasitic Diseases with Clinic, Faculty of Veterinary Medicine, University of Zagreb, Zagreb, Croatia; 30 Parasitological Laboratory, Department of Microbiology, Clinical Center of Serbia, Belgrade, Serbia; 31 Department of Veterinary Public Health and Food Safety, Faculty of Veterinary Medicine, Ghent University, Salisburylaan 133, 9820 Merelbeke, Belgium; 32 Laboratory of Parasitology, Faculty of Veterinary Medicine, Ghent University, Salisburylaan 133, B-9820 Merelbeke, Belgium; 33 Department of Epidemiology and Public Health, Sciensano, Brussels, Belgium

**Keywords:** *Taenia solium*, *Taenia saginata*, Epidemiology, Bovine, Porcine, Neurocysticercosis, Eastern Europe

## Abstract

**Background:**

*Taenia solium* and *Taenia saginata* are food-borne parasites of global importance. In eastern Europe only fragmented information is available on the epidemiology of these zoonotic parasites in humans and animal populations. In particular for *T. solium*, on-going transmission is suspected. The aim of this systematic review was to collect the available data and describe the current knowledge on the epidemiology of *T. solium* and *T. saginata* in eastern Europe.

**Methods:**

Literature published in international databases from 1990 to 2017 was systematically reviewed. Furthermore, local sources and unpublished data from national databases were retrieved from local eastern European experts. The study area included 22 countries.

**Results:**

Researchers from 18 out of the 22 countries provided data from local and unpublished sources, while no contacts could be established with researchers from Belarus, Kosovo, Malta and Ukraine. Taeniosis and human cysticercosis cases were reported in 14 and 15 out of the 22 countries, respectively. Estonia, the Former Yugoslav Republic of Macedonia, Lithuania, Moldova, Poland, Romania, Serbia, and Slovakia reported cases of porcine cysticercosis. Croatia, Czech Republic, Estonia, Former Yugoslav Republic of Macedonia, Moldova, Poland, Romania, Serbia, Slovakia, and Ukraine reported bovine cysticercosis.

**Conclusions:**

There is indication that taeniosis and cysticercosis are present across eastern Europe but information on the occurrence of *T. solium* and *T. saginata* across the region remains incomplete. Available data are scarce and species identification is in most cases absent. Given the public health impact of *T. solium* and the potential economic and trade implications due to *T. saginata,* notification of taeniosis and human cysticercosis should be implemented and surveillance and notification systems in animals should be improved.

**Electronic supplementary material:**

The online version of this article (10.1186/s13071-018-3153-5) contains supplementary material, which is available to authorized users.

## Background

*Taenia solium* and *Taenia saginata*, also referred to as the pork and beef tapeworm, respectively, are food-borne parasites of global importance. In 2014, *T. solium*, for which humans act as final hosts after consumption of undercooked pork, was ranked by an international panel of experts as the food-borne parasite of greatest global concern, affecting millions of individuals every year and causing a substantial economic impact [1]. In 2016, during a similar exercise carried out by a group of experts at the European level, *T. solium* was ranked tenth among 27 parasites as the number of human cysticercosis cases is not as high when Europe is considered as a whole. If individual regions were taken into account, the parasite was ranked higher in eastern Europe than in other parts of Europe [2]. Human cysticercosis is a condition that may arise when humans ingest eggs of *T. solium*. When the parasite migrates to the central nervous system, the neurological condition is referred to as neurocysticercosis (NCC), which may lead to a number of potentially debilitating neurological manifestations including epilepsy and severe progressive headache [3]. NCC is a major global public health concern. Recently, *T. solium* was identified as the number one food-borne parasite contributing to the global burden of disease leading to approximately 28,000 deaths per year and around 2.8 million disability-adjusted life years (DALYs) [4].

*Taenia saginata* ranked lower in the multicriteria-based ranking, as symptoms of taeniosis are mild or even absent and generally the disease is not of substantial public health concern [1, 2]. Nevertheless, the parasite can pose major obstacles for trade and causes a substantial financial burden due to carcass condemnation, freezing and devaluation [5–8].

*Taenia solium* is highly endemic in pork-consuming poor communities of Asia, Africa and Latin America, while *T. saginata* is distributed worldwide. Little is known about the situation of *T. solium* in eastern Europe, and the suspicion of on-going transmission persists [9]. For *T. saginata* a recent review on the epidemiology of bovine cysticercosis revealed the presence of the parasite in a large number of countries in western Europe; however, for eastern Europe data were scarce and of poor quality [10].

In eastern Europe, various socioeconomic and political developments such as the collapse of the Soviet Union and the dissolution of the Socialict Federal Republic of Yugoslavia in 1991 following the wars, led to political and demographic changes. As a result a number of sectors, including the veterinary and public health sectors, were negatively affected. Large numbers of veterinary control officers were replaced by a cheaper working force leading to deteriorated veterinary control systems [11]. The change to small-scale farms with reduced rearing standards and biosecurity and to small abattoirs with insufficient meat inspection and reduced government and veterinary oversight possibly contributed to an increased prevalence of zoonotic parasites such as *T. solium*, *T. saginata*, *Trichinella* spp. and *Toxoplasma gondii* [11]. Backyard slaughtering without meat inspection has also become popular, possibly leading to the perpetuation of zoonotic diseases in parts of the region [12].

Meat inspection is obligatory at slaughterhouses in the European Union (EU), according to European Regulation 854/2004 [13]. Eastern Europe consists of “old” and “new” EU Members States as well as states which are still in the pre-accession phase as candidate EU countries. Therefore, adaptation of the national legislation and/or development of implementing regulations which integrate the main principles and features of the EU legislation and institutional structures towards more modern approaches to achieve better surveillance are ongoing [11, 14].

In the past decade, the number of diagnosed autochthonous NCC cases was increasing in eastern Europe [15–17], leading to the suspicion of on-going *T. solium* transmission occurring in the area; however, according to experts the numbers have gone down in the last few years.

Changes in eating habits might further contribute to the current transmission patterns of the parasite, while imported *T. solium* tapeworm carriers and increased migration and travels further contribute to the risk [18]. More recently, increasingly, European integration and introduction of regulatory legislation have influenced the region and, e.g. made it mandatory to have modern sanitation [11].

Both *T. solium* and *T. saginata* have been considered endemic in eastern Europe. Results reported from the region in the past should however be evaluated critically for their representativeness and for the laboratory procedures applied [14]. In the World Health Organization *T. solium* endemicity map, updated in 2015, uncertainty on the epidemiological situation in eastern Europe persists [19].

In order to advance our knowledge on the occurrence of these zoonotic parasites and assess the potential needs for surveillance, we performed a systematic review on the epidemiology of taeniosis/cysticercosis in eastern Europe. This review is part of a series of two systematic reviews: the first one covered western Europe [10] and the current one covers eastern Europe.

## Methods

We conducted a systematic review following the Preferred Reporting Items for Systematic Reviews and Meta-Analyses (PRISMA) guidelines [20] (Additional file 1). To search for information on the epidemiology of *T. saginata* and *T. solium* in eastern Europe we used international databases and local, unpublished sources. Epidemiology was defined as the occurrence, prevalence, incidence and geographical distribution of human/porcine/bovine cysticercosis and taeniosis. Eastern Europe was defined based on regional proximity and on gross domestic product/gross national income and included 22 countries: Albania, Belarus, Bosnia and Herzegovina, Bulgaria, Croatia, Cyprus, Czech Republic, Estonia, Former Yugoslav Republic of Macedonia, Greece, Hungary, Kosovo, Latvia, Lithuania, Malta, Moldova, Montenegro, Poland, Romania, Serbia, Slovakia and Ukraine [10].

### International databases

The online international bibliographical databases PubMed, ISI Web of Knowledge, CABDirect, OAIster, and OpenGrey were searched for all published data on taeniosis and (neuro)cysticercosis using the following search phrase: (cysticerc* OR cisticerc* OR neurocysticerc* OR neurocisticerc* OR "C. bovis" OR "C. cellulosae" OR taenia* OR tenia* OR saginata OR solium OR taeniosis OR teniosis OR ténia OR taeniid OR cysticerque) AND (Albania OR Belarus OR Bosnia OR Herzegovina OR Bulgaria OR Croatia OR Cyprus OR Czech Republic OR Estonia OR Former Yugoslav Republic of Macedonia OR Greece OR Hungary OR Kosovo OR Latvia OR Lithuania OR Malta OR Moldova OR Montenegro OR Poland OR Romania OR Serbia OR Slovakia OR Ukraine). The databases were searched for papers published from January 1st 1990 up to January 31st 2017. No language restriction was applied.

Papers were excluded when fulfilling one or more of the following criteria: (i) studies concerning a different parasite than *T. saginata* and/or *T. solium*; (ii) studies reporting data from outside the specified countries; (iii) studies reporting results out of the scope of the review questions; (iv) duplicates. Papers were first screened for eligibility based upon title and abstract, and in the case of doubt, the full paper was assessed. For each eligible document, a narrative synthesis was made, which were then further digested into a qualitative review.

### Local sources

To identify additional, locally published and unpublished data sources, we distributed country sheets (Additional file 2) to members of the European Network on Taeniosis/Cysticercosis (CYSTINET) COST Action TD1302 and other non-member experts, asking to specify relevant national journals or epidemiological bulletins, MSc/PhD dissertations, national institutes, or registries, and to translate relevant search terms. Due to ethical constraints, unpublished hospital or laboratory data were only presented at an aggregated level. In addition, we searched for information in the proceedings of the CYSTINET (European Network for taeniosis/cysticercosis, COST Action TD1302) and European Network for Foodborne Parasites (COST Action FA1408, Euro-FBP) meetings. Finally, we explored the reference lists of recent topic-specific reviews [15–17] to find additional eligible papers. We applied the same inclusion and exclusion criteria to these additional sources and developed narrative syntheses for all eligible documents.

### Data collection and analyses

The data collection was performed by three independent reviewers (CT, BD and SS). For data analysis, we followed the methodology described in [10]. Briefly, cases reported as case reports providing information on individual characteristics of the patient were defined as individual cases and when no individual information was given, these cases were defined as aggregated. Tables summarizing individual cases included year of diagnosis, age, gender, nationality, and reported risk factors, while for aggregated cases or prevalence, the tables included country, level of data collection, timeframe, number of cases (or prevalence or incidence), *Taenia* species, risk factors (e.g. immigration/travel history) if available, and reference (i.e. authors and publication year).

The following definitions were applied for the description of risk factors: endemic region (Asia, Africa and South and Central America, including Caribbean islands); immigrant from endemic region (any person born in or native from endemic region, or reported to have moved from endemic region); travelled/stayed in endemic region (having travelled, stayed, or resided in endemic region); no history of travels to endemic areas or immigration (autochthonous) (no history of travel/immigration reported).

Cases in which the existence of duplicates was probable (e.g. cases included in two retrospective studies on the same area/hospital, covering overlapping time periods, cases diagnosed in the same hospital in the same time-frame but reported in different sources etc.) were included only once, by selecting the one that was published first.

Descriptive analyses and maps were performed using the software environment for statistical computing R 3.5.0 and graphs were made in both R and Microsoft Excel [21].

## Results

### Search results

**Fig. 1:**
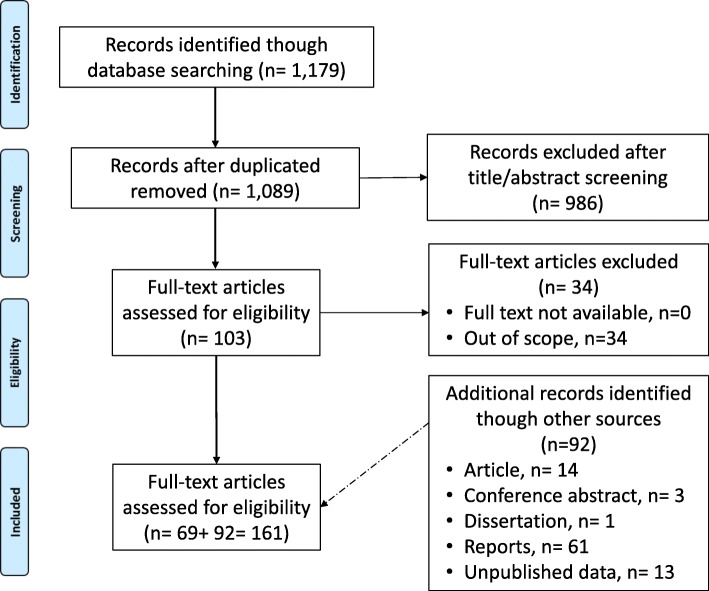
Flow diagram of the search strategy steps

**Fig. 2:**
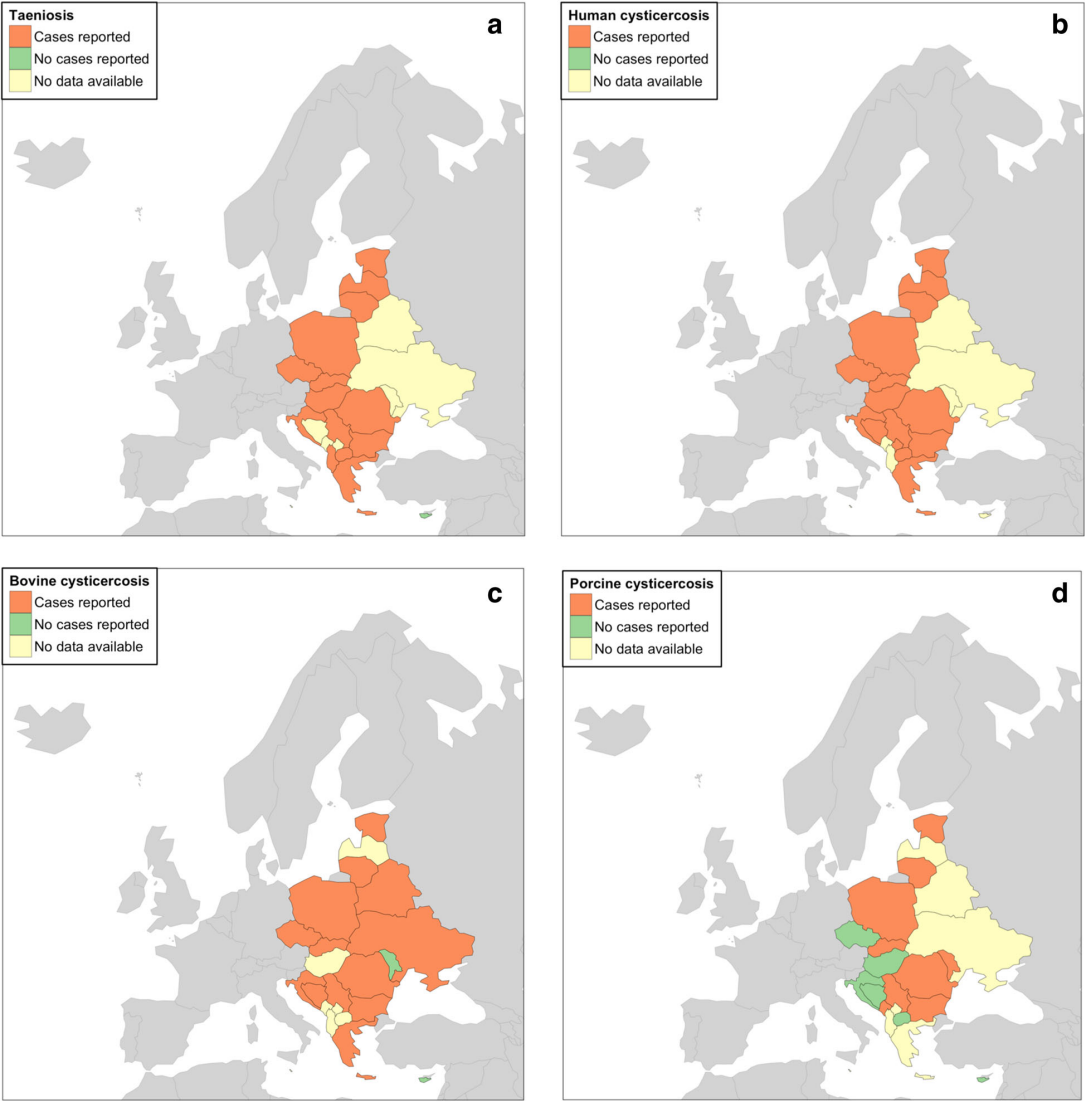
Summary of identified data on taeniosis and cysticercosis (in humans and animals) in eastern Europe (1990–2017): taeniosis (**a**); human cysticercosis (**b**); bovine cysticercosis (**c**); and porcine cysticercosis (**d**)

The initial search resulted in 1179 peer reviewed papers identified though international databases. After the screening process, 69 relevant references were identified and included in the review (Additional file 3: Table S1). In addition to this, researchers of 18 out of 22 eastern European countries were contacted from which additional data (92 relevant sources) from local and unpublished sources were obtained (Additional file 4: Table S2). Contacts of researchers working within the field from Belarus, Kosovo, Malta and Ukraine were not available; hence local, unpublished data for these countries are missing. The flow diagram of the search strategy steps is presented in Fig. 1. The countries for which data on *T. solium* or *T. saginata* in humans and/or animals were found are presented in Fig. 2.

### Taeniosis

In total, 58 unique sources were identified providing information on taeniosis in eastern Europe. Individual (8 records) and/or aggregated cases/prevalence (50 records) of taeniosis were reported in 14 out of 22 eastern European countries (Fig. 2).

No cases of taeniosis were reported in Cyprus, and for 7 countries (Belarus, Bosnia and Herzegovina, Kosovo, Malta, Moldova, Montenegro and Ukraine), no reports on taeniosis could be retrieved for the years between 1990 and 2017. Poland was the country recording most data on taeniosis.

### Taeniosis case reports

In total only 17 individual cases reported in case reports from Croatia, Greece, Lithuania and Serbia, were available (Additional file 5: Table S3). Out of the 17 cases, 13 were reported as *Taenia* spp., three reported as confirmed and one suspected *T. saginata* using microscopy.

No case reports describing *T. solium* taeniosis cases were identified through the search of published and other unpublished grey literature. Moreover none of all the case reports reported possible risk factors or how the parasite infection was acquired.

### Aggregated taeniosis cases

**Fig. 3:**
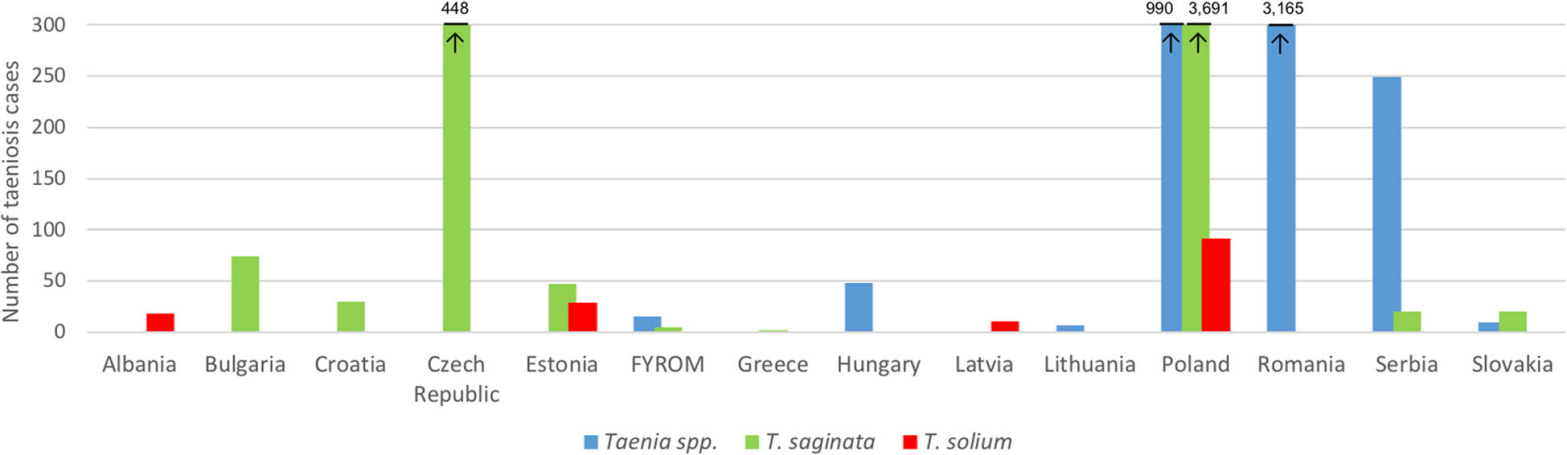
Taeniosis cases reported in scientific publications, authority reports, epidemiologial bulletins, and laboratories in eastern Europe (published/reported between 1990–2017). Arrow pointing at the number, larger than 300 taeniosis cases. *Abbreviation*: FYROM, Former Yugoslav Republic of Macedonia

Data on aggregated taeniosis cases were obtained from scientific publications, authorities’ reports, national registries, epidemiological bulletins, and from hospitals/laboratories (Additional file 5: Table S4). Aggregated taeniosis cases were reported in 14 eastern European countries and covered different years. Data were obtained from publications ranging from the 1990 up to 2017 (Fig. 3). The largest proportion of cases was reported as *Taenia* spp. and at species level the majority of cases were reported as *T. saginata*. *Taenia solium* taeniosis cases were a rare finding, reported only in Albania, Estonia, Latvia and Poland. No indication was given on how species identification was performed. For Latvia, the two suspected cases probably originated from abroad (probably Russia) (Deksne, personal communication, 2016). No additional information on the source of infection, potential risk factors or nationality was given for the other reported cases.

The annual number of cases reported for each country varied, with Poland and Romania reporting the highest annual number of aggregated cases. In particular for Poland, a large number of data was available as epidemiological reviews published quarterly in the journal of the National Institute of Public Health - National Institute of Hygiene and the Polish Society of Epidemiology and Infectious Diseases.

### Taeniosis incidence data

**Fig. 4:**
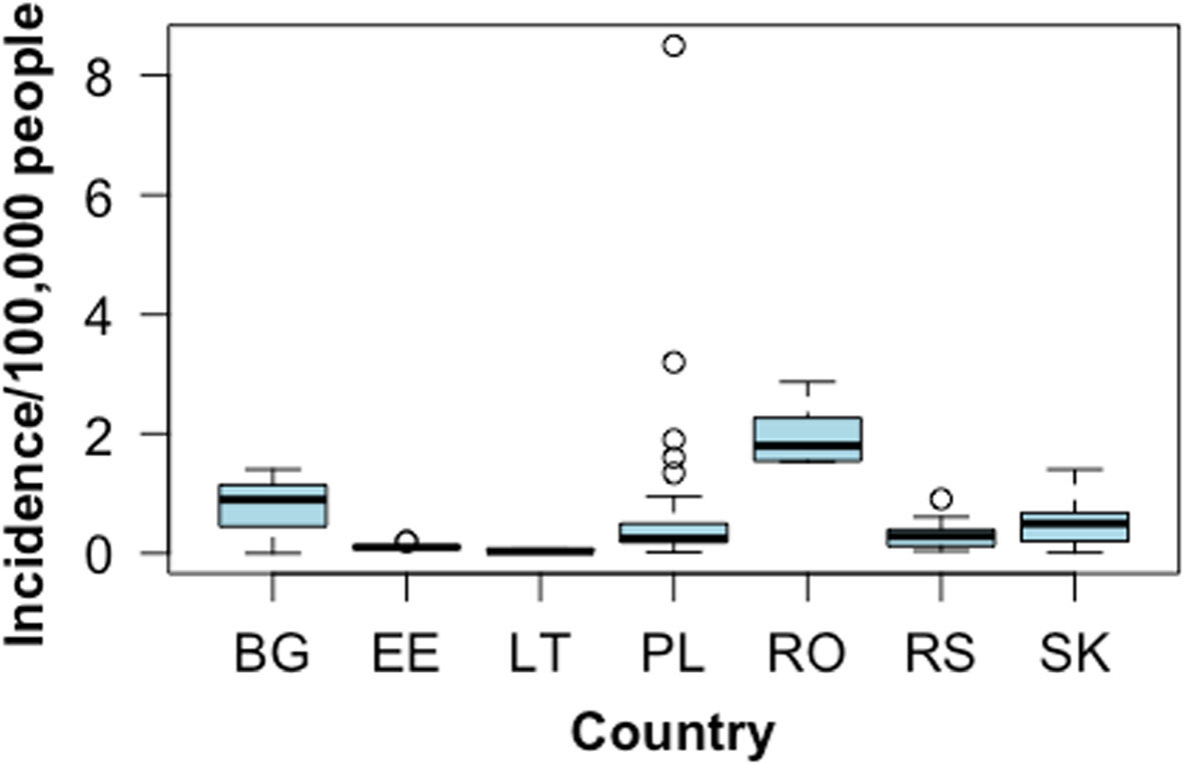
Taeniosis incidence data reported between the years 1990–2017 in authorities’ reports, epidemiological bulletins and national registries in eastern Europe. *Abbreviations*: BG, Bulgaria (1998 and 2000); EE, Estonia (1990–1999); PL, Poland (1991–2009); RO, Romania (2007–2014); RS, Serbia (1997–2005); SK, Slovakia (1990–2014)

Incidence data were available for seven counties (Bulgaria, Estonia, Lithuania, Poland, Romania, Serbia and Slovakia) and ranged from 0/100,000 people for Bulgaria in the year 2000 to 8.5/100,000 people in Poland in 1991 (Fig. 4).

### Taeniosis prevalence data

In total, five epidemiological studies reporting taeniosis prevalence data published between 1990**–**2017 were identified (Additional file 5: Table S5). Within eastern Europe the prevalence of taeniosis ranged between 0–4.9%, with the highest prevalence reported in a study conducted in children of the Roma settlements of Košice and Prešov regions in Slovakia [22]. A study carried out in Estonia reported a seroprevalence of *T. solium* of 0.7% (95% confidence interval, CI: 0.3–1.4%) using a commercial NovaLisa IgG enzyme immunoassay ELISA [NovaTec Immunodiagnostica GmbH, Dietzenbach, Germany (Sp > 95%, Se 94%)], and 0.0% (95% CI: 0–0.3%) using the ELISA and a Western Blot IgG (LDBIO DIAGNOSTICS, Lyon, France) in series [23], while the other four studies used microscopy as a diagnostic tool.

### Human cysticercosis

Relevant information on human cysticercosis was obtained from 63 sources. In total, 40 reports reported individual, and 23 reports reported aggregated cysticercosis cases. Information on human cysticercosis cases was available for 15 out of 22 eastern European countries (Fig. 2), while no data could be retrieved from Albania, Belarus, Cyprus, Malta, Moldova, Montenegro and Ukraine in the form of either published or unpublished sources.

### Human cysticercosis case reports

In total, 58 individual cases of cysticercosis were identified in authorities’ reports, from hospital/laboratories or published case reports (Table 1). Out of 58 cases, 45 were diagnosed in 7 eastern European countries, while 13 cases (22%) were originally from eastern Europe but were diagnosed abroad. The average age was 49 years and 49% of the cases were male, 42% female and for 9% the sex was not known (Additional file 5: Table S6). Of all the individual case reports, 17% were reportedly suspected to be autochthonous cases, in 14% the infection was considered aquired in endemic countries while travelling or living abroad, whereas for the majority of cases (69%) the place of infection was unknown or not recorded. For Lithuania, the cases were possibly connected with travels to Argentina and South Africa (S. Petkevičius, personal communication, 2015).

**Table 1:** Number of human cysticercosis cases per country and most likely place of infection (Data from published and unpublished sources between 1990 and 2017)

Country	Most likely place of infection			Total no. of cases
	Eastern Europe	Endemic country	Unknown	
Croatia			10	10
Czech Republic	6	4		10
Greece	3	3	6	12
Hungary			2	2
Latvia		1	1	2
Lithuania			5	5
Serbia	1		5	6
Cyprus			1	1
Czechoslovakia			2	2
Former Yugoslav Republic of Macedonia			3	3
Bosnia and Herzegovina			5	5

### Aggregated human cysticercosis cases

**Fig. 5:**
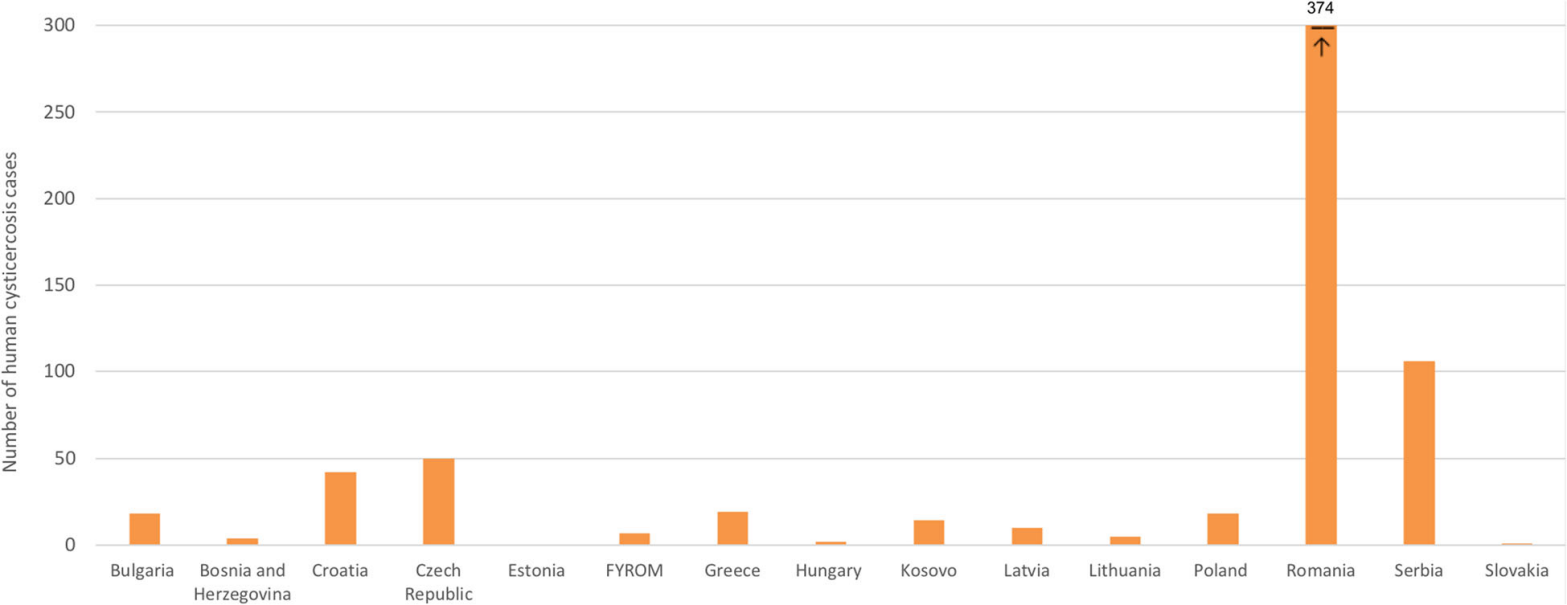
Number of identified human cysticercosis cases in eastern Europe - data sources published and unpublished between 1990–2017. Arrow pointing at the number, larger than 300 human cycsticercosis cases. *Abbreviation*: FYROM, Former Yugoslav Republic of Macedonia

The number of aggregated human cysticercosis cases varied largely across countries, with over 300 cases (most of them reported as autochthonous) reported from Romania. Figure 5 shows the number of human cysticercosis cases identified in eastern Europe in documents published from 1990 to 2017. Information on risk factors and place where the disease might have been acquired were absent for most cases (Additional file 5: Table S7).

Only two serological studies using ELISA and/or western blot techniques were identified. One of the studies was in Croatia, where a prevalence of 1.5% was recorded among people with epilepsy [24]. The other study was in Estonia, where a western-blot-confirmed *T. solium* cysticercosis prevalence (commercial ELISA and commercial western blot in series) was of 0.0% in samples from the general Estonian population, children, veterinarians and hunters [23].

### Porcine cysticercosis

Relevant information on porcine cysticercosis was obtained from 53 sources, which reported the occurrence of the infection in 8 countries. In Poland the causative agent was specified as being “*cysticercus cellulosae*” (name used for *T. solium* cysticercus) in two publications (Additional file 5: Table S8)*.* The highest number of porcine cysticercosis cases (4487 cases) was recorded in Poland in 2003; however, in the report species were not specified and porcine cysticercosis could refer to “*cysticercus cellulosae*” or cysticerci of other species like “*cysticercus tenuicollis*” (name used for *Taenia hydatigena* cysticercus). Of the porcine cysticercosis cases reported, a molecular method was reportedly used only in Estonia, and *T. solium* could not be confirmed [25]. No cases of porcine cysticercosis in pigs were reported from Bosnia and Herzegovina, Croatia, Cyprus, Czech Republic, Former Yugoslav Republic of Macedonia and Hungary, and no information was found for Albania, Belarus, Greece, Kosovo, Malta and Ukraine. Based on routine meat inspection, reported prevalence ranged from 0% to 0.18% in Bulgaria [9] and varied within and between countries. A single serological study was identified: in Romania, an ELISA-based prevalence of active porcine cysticercosis was reported to be 6.4% [26].

In Latvia, according to the Food and Veterinary Service, the main slaughtering monitoring authority, in the last 10 years no suspicious cysts have been found in pigs (Food and Veterinary Service Latvia, personal communication, 2015).

### Bovine cysticercosis

**Fig. 6:**
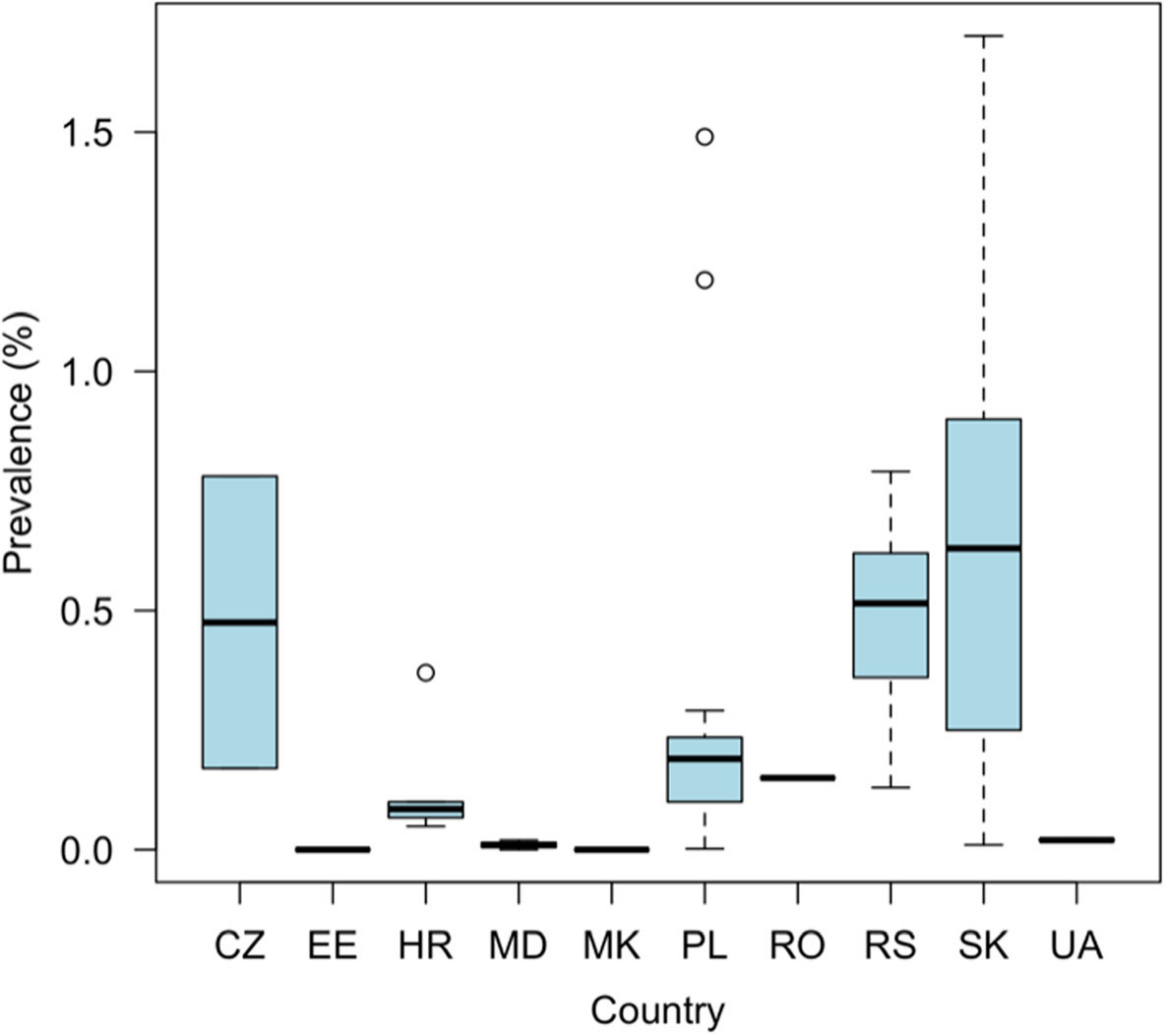
Prevalence of bovine cysticercosis based on routine meat inspection detected in eastern Europe (1990–2017). *Abbreviations*: CZ, Czech Republic; EE, Estonia; HR, Croatia; MD, Moldova; MK, Former Yugoslav Republic of Macedonia; PL, Poland; RO, Romania; RS, Serbia; SK, Slovakia; UA, Ukraine

Relevant information on bovine cysticercosis was obtained from 41 sources reporting data for 15 out of 22 countries (Additional file 5: Table S9). Only for Cyprus, no cases were recorded since 1990. For Albania, Hungary, Kosovo, Latvia, Malta and Montenegro, no data were retrieved. Based on routine meat inspection, reported prevalence ranged from 0.0% up to 1.7% and varied within and between countries (Fig. 6). No studies were found applying more sensitive methods to detect bovine cysticercosis. For Poland in particular, a large amount of data were available, in which the highest annual number of bovine cysticercosis cases (4718 cases) in the country was reported in 1994. The number of reported cases has decreased consistently over the years.

## Discussion

Published and unpublished data sources were searched, and contacts with researchers of eastern European countries were established to obtain all possible information on the epidemiology of *T. solium* and *T. saginata* in eastern Europe.

Individual case reports for taeniosis were only retrieved from four countries, while reports on aggregated cases were available for nearly all the eastern European countries. At a species level, most data were reported as *Taenia* spp. or *T. saginata*, leading to uncertainty regarding the true disease epidemiology. In a few countries, *T. solium* was reported as aggregated taeniosis cases. Given the risk of having *T. solium* carriers it is essential to improve on differential diagnosis and reporting. A similar scenario was also observed in western Europe, suggesting that diagnosis and case management is not performed adequately [10]. Moreover, the laboratory procedures and the reporting seem unstandardized and often only a small part of the population is examined; therefore, the data from most countries are incomplete [14]. Hence, the reported prevalence of human taeniosis from *T. saginata* and *T. solium* should be evaluated critically. Also, the origin of infection and risk factors were not reported, leading to continued uncertainty on the real endemic status of eastern Europe.

Human cysticercosis reports of individual and aggregated cases were available from nearly one third of the 22 eastern European countries, while for western Europe, information was available from all 18 countries [10]. Romania and Serbia reported the largest number of diagnosed human cysticercosis cases, highlighting both the reporting and possibly also the epidemiological differences among eastern European countries. Observing the difference in reported cases at a hospital level between countries, either an underestimation or an overestimation might be suspected for eastern Europe. However, several factors such as lack of knowledge, diagnostic capacity and compensation for the farmers, unwillingness or inability to report and logistic difficulties may contribute to the vicious cycle of underreporting and underdiagnosis [27, 28]. Of all cases, 14% were suspected to have travelled abroad, 17% to be autochthonous cases, while for the majority (69%) it was not specified. This lack of additional information does not allow us to unravel the question of an existing parasite transmission in eastern Europe. The results show that even if the assumption that the infection can be acquired abroad would be correct, then the same happens in Europe as frequently. The automatic assumption that brief visits abroad automatically outrule looking for answers locally, can lead to a barrier for dealing with the actual epidemiological situation.

Nearly one quarter of the cases were diagnosed abroad, possibly indicating that either the local health system is not equipped to detect these cases, or that people are actively seeking medical attention elsewhere.

For two thirds of the human cases the origin of infection was not given, further contributing to the persisting knowledge and underreporting gap. However, it should be noted that a lowering trend is registered. For instance in Serbia, no new cases were recorded after 2010.

Cases of porcine cysticercosis were reported in a number of countries, and as for the human cases, the number of cases and reports per country varied significantly. Notably, in Poland over 4000 cases of porcine cysticercosis were recorded by the veterinary inspection services in 2003; however, no information on the *Taenia* spp. involved was given and the possibility of (some of) the cases being *T. hydatigena* cannot be excluded. Also, for most other porcine cysticercosis cases reported in eastern Europe, species identification was never performed, nor molecularly confirmed. Only in one study from Estonia, molecular techniques were applied to confirm a false positive case [25]. Lack of differential diagnosis highlights the presence of poor meat inspection, due to the possible lack of: qualified meat inspectors, sensitive and specific diagnostic tools, awareness, funding and adequate mandatory reporting systems. Moreover the trend towards smaller-scale pig farming, with outdoor access and home slaughtering (without meat inspection) increases the risk of infection and therefore the need for monitoring.

Bovine cysticercosis was reported in over two thirds of the countries in the region. The highest number of cases was reported in Poland, where 4718 bovine cysticercosis cases were recorded in 1994. General meat inspection has a very low sensitivity, hence slaughterhouse figures, even when well-recorded will always underestimate the prevalence [29]. False positives, e.g. eosinophilic myositis as a consequence of sarcocystis infection or abscessess, can also occur although they usually contribute only a small (negligible) proportion of bovine cysticercosis records.

Better, cost-effective and more sensitive diagnostic tools should be developed, and data recording systems should be improved to assure that data from individual slaughterhouses are centralised. Increased availability of data will further allow estimating the overall economic impact of *T. saginata* which has been shown to be significant in Belgium (858 €/heavily infected carcass and 586 €/lightly infected carcass) [7], but less so in Catalonia [northeastern Spain (1140 €/heavily infected carcass and 509 €/lightly infected carcass)] [8]. Without reliable data from the surveillance the default assumption should be that the parasite is present, not absent. To overcome case uncertainty and to be able to have more accurate data that will improve our knowledge on the epidemiological situation of *T. saginata* and *T. solium*, the possibility to molecularly confirm *T. solium* findings should be available. Recently, the laboratories across Europe that are running tests for the larval and adult stages of the parasites have been summarised and mapped, facilitating the process when suspected lesions are detected [27].

The early species-level identification of the tapeworm and subsequent adapted management are crucial to avoid not only human-to-human transmission, but also human-to-pig/cattle transmission.

Finally, for nearly all the countries, at least some data and research on the topic could be retrieved and both published and unpublished data sources were available. Particularly the unpublished sources were of added value and contributed to an improved picture of the epidemiological situation in eastern Europe, highlighting the importance of data access and collaborations through networks such as CYSTINET. Nevertheless, for Belarus, Kosovo, Malta and Ukraine a contact could not be established and no information could be retrieved leaving information gaps. In the absence of data the presence of *T. saginata* and *T. solium* in these countries cannot be excluded. For western Europe information and a contact was established with all counties of the region, and the amount of published and unpublished literature was more than double that of eastern Europe, which helped to provide a better and more complete overview of the epidemiological situation [10]. The two zoonotic parasites would merit more attention and research in eastern Europe.

## Conclusions

There is indication that taeniosis and cysticercosis are present across eastern Europe but information on the occurrence of *T. solium* and *T. saginata* across the region remains fragmented. For a number of countries, no information was available and for some, establishing a contact with experts within the field proved challenging. Species identification is not performed in most countries and the findings are not confirmed using molecular methods (especially for taeniosis and porcine cysticercosis), making the endemicity status in the region still unclear. Attention should be paid to: (i) suspected autochthonous human cysticercosis cases to rule out ongoing transmission; (ii) notification of taeniosis and human cysticercosis as these should be implemented to get better data and a clearer overview of the epidemiological situation; (iii) surveillance and reporting systems in animals; and (iv) methods for confirming findings.

## Additional files

Additional file 1: PRISMA checklist. (PDF 68 kb)

Additional file 2: Country sheets template. (PDF 81 kb)

Additional file 3: **Table S1.** List of references included in the review retrieved through online international databases. (XLSX 24 kb)

Additional file 4: **Table S2.** List of references included in the review made available through local sources. (XLSX 71 kb)

Additional file 5: **Table S3.** Individual taeniosis cases identified in case reports in eastern Europe available from 1990 to 2017. **Table S4.** Aggregated taeniosis cases reported in documents (authorities’ reports, epidemiological bulletins, national registries and publications) identified in eastern Europe available from 1990 to 2017. **Table S5.** Taeniosis prevalence data reported in epidemiological studies published between 1990–2017. **Table S6.** Individual human cysticercosis cases identified in case reports in eastern Europe (1990–2017). **Table S7.** Aggregated human cysticercosis cases identified in case reports and publications in eastern Europe (1990–2017). **Table S8.** Porcine cysticercosis cases identified and reported during meat inspection in case reports and publications in eastern Europe (1990–2017). **Table S9.** Bovine cysticercosis cases identified and reported during meat inspection in case reports and publications in eastern Europe (1990–2017). (DOCX 207 kb)

## Abbreviations

CYSTINET: European Network on Taeniosis/Cysticercosis
EFSA: European Food Safety Authority
GDP/GNI: gross domestic product/gross national income
ICD: International Classification of Diseases
NCC: neurocysticercosis
OIE: World Organization for Animal Health/Office International des Epizooties
