# Correction to: Epidemiology of taeniosis/cysticercosis in Europe, a systematic review: eastern Europe

**DOI:** 10.1186/s13071-019-3328-8

**Published:** 2019-02-18

**Authors:** Chiara Trevisan, Smaragda Sotiraki, Minerva Laranjo-González, Veronique Dermauw, Ziqi Wang, Age Kärssin, Aleksandar Cvetkovikj, Andrea S. Winkler, Annette Abraham, Branko Bobić, Brian Lassen, Carmen Michaela Cretu, Cozma Vasile, Dimitris Arvanitis, Gunita Deksne, Ilievski Boro, István Kucsera, Jacek Karamon, Jovana Stefanovska, Břetislav Koudela, Maja Jurhar Pavlova, Marian Varady, Marina Pavlak, Mindaugas Šarkūnas, Miriam Kaminski, Olgica Djurković-Djaković, Pikka Jokelainen, Dagny Stojčević Jan, Veronika Schmidt, Zorica Dakić, Sarah Gabrië, Pierre Dorny, Brecht Devleesschauwer

**Affiliations:** 1 Department of Biomedical Sciences, Institute of Tropical Medicine, Nationalestraat 155, 2000 Antwerp, Belgium; 2 Department of Veterinary and Animal Sciences, Faculty of Health and Medical Sciences, University of Copenhagen, Dyrlægevej, 100 Frederiksberg, Denmark; 3 Veterinary Research Institute, HAO-DEMETER, Campus Thermi, 57001 Thessaloniki, Greece; 4 IRTA, Centre de Recerca en Sanitat Animal (CReSA, IRTA-UAB), Campus de la Universitat Autònoma de Barcelona, 08193 Bellaterra, Spain; 5 University of Florida College of Medicine, Gainesville, Florida, USA; 6 Veterinary and Food laboratory, Kreutzwaldi 30, 51006 Tartu, Estonia; 7 Institute of Veterinary Medicine and Animal Sciences, Estonian University of Life Sciences, Kreutzwaldi 1, 51006 Tartu, Estonia; 8 Department of Parasitology and Parasitic Diseases, Faculty of Veterinary Medicine, Ss. Cyril and Methodius University in Skopje, Lazar Pop Trajkov 5–7, 1000 Skopje, Former Yugoslav Republic of Macedonia; 9 Centre for Global Health, Department of Neurology, Technical University Munich, Ismaninger Strasse 22, 81675 Munich, Germany; 10 Centre for Global Health, Department of Community Medicine and Global Health, Institute of Health and Society, University of Oslo, Kirkeveien 166, 0450 Oslo, Norway; 11 Centre of Excellence for Food- and Vector-Borne Zoonoses, Institute for Medical Research, University of Belgrade, Belgrade, Serbia; 12 Department of Parasitology, Carol Davila University of Medicine and Pharmacy, Colentina Clinical Hospital, Bucharest, Romania; 13 Department of Parasitology, University of Agricultural Sciences and Veterinary Medicine Cluj-Napoca, Cluj-Napoca, Romania; 14 Department of Microbiology, 424 Military General Hospital, Thessaloniki, Greece; 15 Institute of Food Safety, Health and Environment, Riga, Latvia; 16 Faculty of Biology, University of Latvia, Riga, Latvia; 17 Institute for Pathology, Medical Faculty, University “Ss. Cyril and Methodius”, Skopje, Former Yugoslav Republic of Macedonia; 18 Department of Parasitology, National Institute for Public Health, Budapest, Hungary; 19 Department of Parasitology and Invasive Diseases, National Veterinary Research Institute in Pulawy, Pulawy, Poland; 20 Department of Pathology and Parasitology, Faculty of Veterinary Medicine, University of Veterinary and Pharmaceutical Sciences Brno, Palackého tř. 1946/1, 61242 Brno, Czech Republic; 21 Central European Institute of Technology, University Trevisan et al. Parasites & Vectors (2018) 11:569 Page 10 of 11 of Veterinary and Pharmaceutical Sciences Brno, Palackého tř. 1946/1, 61242 Brno, Czech Republic; 22 Institute for Microbiology and Parasitology, Medical faculty, University “Ss. Cyril and Methodius”, Skopje, Former Yugoslav Republic of Macedonia; 23 Institute of Parasitology, Slovak Academy of Sciences, Košice, Slovakia; 24 Department of Veterinary Economics and Epidemiology, Faculty of Veterinary Medicine, University of Zagreb, Zagreb, Croatia; 25 Lithuanian University of Health Sciences, Kaunas, Lithuania; 26 Department of Neurology, Klinikum rechts der Isar, Technical University Munich, Ismaninger Straße 22, 81675 Munich, Germany; 27 Department of Bacteria, Laboratory of Parasitology, Parasites and Fungi, Infectious Disease Preparedness, Statens Serum Institute, Copenhagen, Denmark; 28 Faculty of Veterinary Medicine, University of Helsinki, Helsinki, Finland; 29 Department of Parasitology and Parasitic Diseases with Clinic, Faculty of Veterinary Medicine, University of Zagreb, Zagreb, Croatia; 30 Department of Microbiology, Parasitological Laboratory, Clinical Center of Serbia, Belgrade, Serbia; 31 Department of Veterinary Public Health and Food Safety, Faculty of Veterinary Medicine, Ghent University, Salisburylaan 133, 9820 Merelbeke, Belgium; 32 Laboratory of Parasitology, Faculty of Veterinary Medicine, Ghent University, Salisburylaan 133, B-9820 Merelbeke, Belgium; 33 Department of Epidemiology and Public Health, Sciensano, Brussels, Belgium

**Correction to: Parasit Vectors (2018) 11:569**

**https://doi.org/10.1186/s13071-018-3153-5**

In the original article [1], the authors Dr Jasmin OMERAGIĆ and Dr Davor ALAGIĆ were erroneously omitted from the co-authors list. The authors significantly contributed to the article by providing data for Bosnia and Herzegovina, and by reading and approving the final version of the manuscript. As such, the authors have now been added to the original article’s [1] co-authors list.
