# Supplementary material for: Epidemiology of taeniosis/cysticercosis in Europe, a systematic review: eastern Europe
Source: Parasit Vectors. 2018 Oct 30;11:569. doi: 10.1186/s13071-018-3153-5 (PMC6208121; doi:10.1186/s13071-018-3153-5)
Supplement: Supplementary file 2 — Country sheets template. (PDF 81 kb) [file 13071_2018_3153_MOESM2_ESM.pdf]

CYSTINET Country Study Report

Dear colleague,

We are currently looking into the epidemiology of *Taenia solium* and *Taenia saginata* in Europe by means of **Country Studies**.

In order to easily access "sleeping data", we would kindly like to ask you to fill out the tables below to help us **facilitating the search** and **gain access to databases unknown to us**.

We plan to publish the results of these CYSTINET country studies as reviews on the epidemiology of taeniosis/cysticercosis in Europe. We would like to offer authorship to everyone who offered information or data and provided feedback on the manuscript drafts.

We are very grateful for your precious help.

Thank you,

Member of CYSTINET Work group 1.

For further information on the COUNTRY STUDIES, please feel free to contact us:

Your name — your email

Brecht Devleesschauwer — brechtdv@gmail.com

For general information on CYSTINET, please visit http://www.cystinet.org

CYSTINET

cost
EUROPEAN COOPERATION
IN SCIENCE AND TECHNOLOGY

Key words

Please translate the following key words into the main language(s) of your country. Feel free to add more columns if needed. Please replace <Language 1>, etc, by the name of the concerned language.

**Key word**	**<Language 1>**	**<Language 2>**
Tapeworm		
Taenia		
Cysticercus/cysticerc		
Taeniosis		
Cysticercosis		
Neurocysticercosis		

National journals or (sub)national Epidemiological bulletins

Please provide a list of (sub)national journals that may contain information on human and/or animal taeniosis/cysticercosis. Any journal is useful, even if it does not contain English articles. Feel free to use as many rows as needed.

**Journal name/Epidemiological bulletin**	**URL (if available)**

MSc/PhD dissertation databases

Please provide a list of MSc/PhD dissertation databases that may contain information on human and/or animal taeniosis/cysticercosis. The dissertations should not necessarily be in English. Feel free to use as many rows as needed.

**University**	**URL**

National institutes

Please provide the names and websites of the (sub)national institutes that are responsible for the concerned activities. Feel free to duplicate rows if needed.

**Activity**	**Institute/Network**	**URL**
Meat inspection		
Surveillance of infectious diseases in humans		
Surveillance of infectious diseases in animals		

Registries

Please provide the names and websites of (sub)national registries or information networks for these diseases, if they exist. If such registries do not exist, please write “DOES NOT EXIST”.

Disease	Institute/Network	URL
Taeniosis		
Human cysticercosis		
Bovine cysticercosis		
Porcine cysticercosis		
