# Supplementary material for: Epidemiology of taeniosis/cysticercosis in Europe, a systematic review: eastern Europe
Source: Parasit Vectors. 2018 Oct 30;11:569. doi: 10.1186/s13071-018-3153-5 (PMC6208121; doi:10.1186/s13071-018-3153-5)
Supplement: Supplementary file 5 — Table S3. Individual taeniosis cases identified in case reports in eastern Europe available from 1990 to 2017. Table S4. Aggregated taeniosis cases reported in documents (authorities’ reports, epidemiological bulletins, national registries and publications) identified in eastern Europe available from 1990 to 2017. Table S5. Taeniosis prevalence data reported in epidemiological studies published between 1990–2017. Table S6. Individual human cysticercosis cases identified in case reports in eastern Europe (1990–2017). Table S7. Aggregated human cysticercosis cases identified in case reports and publications in eastern Europe (1990–2017). Table S8. Porcine cysticercosis cases identified and reported during meat inspection in case reports and publications in eastern Europe (1990–2017). Table S9. Bovine cysticercosis cases identified and reported during meat inspection in case reports and publications in eastern Europe (1990–2017). (DOCX 207 kb) [file 13071_2018_3153_MOESM5_ESM.docx]

**Additional file 5: Table S3.** Individual taeniosis cases identified in case reports in eastern Europe available from 1990-2017.

| **Country of diagnosis** |  | **Age at diagnosis (years)** | **Gender** | **Country of origin** | ***Taenia* species reported as** | **First author and year** | **Reference** |
| --- | --- | --- | --- | --- | --- | --- | --- |
| Croatia | Unknown | 50 | Female | Unknown | *T. saginata* | Fabijanic et al., 2001 | [1] |
| Greece | Unknown | 28 | Male | Unknown | *T. saginata* | Karanikas et al., 2007 | [2] |
| Greece | Unknown | 76 | Male | Unknown | *T. saginata (suspected)* | Karanikas et al., 2007 | [2] |
| Lithuania | 1996 | Unknown | Unknown | Lithuania | *Taenia spp.* | Lithuanian Centre for Infectious Diseases and HIV. Annual Reports. Vilnius. 1996 |  |
| Lithuania | 2002 | Unknown | Unknown | Lithuania | *Taenia spp.* | Lithuanian Centre for Infectious Diseases and HIV. Annual Reports. Vilnius. 2002 |  |
| Lithuania | 2005 | 0-17 | Female | Lithuania | *Taenia spp.* | Lithuanian Centre for Infectious Diseases and HIV. Annual Reports. Vilnius. 2005 |  |
| Lithuania | 2014 | 18-24 | Female | Lithuania | *Taenia spp.* | Lithuanian Centre for Infectious Diseases and HIV. Annual Reports. Vilnius. 2014 |  |
| Lithuania | 2014 | 35-44 | Female | Lithuania | *Taenia spp.* | Lithuanian Centre for Infectious Diseases and HIV. Annual Reports. Vilnius. 2014 |  |
| Lithuania | 2016 | 0-17 | Male | Lithuania | *Taenia spp.* | Lithuanian Centre for Infectious Diseases and HIV. Annual Reports. Vilnius. 2016 |  |
| Lithuania | 2016 | 55-65 | Male | Lithuania | *Taenia spp.* | Lithuanian Centre for Infectious Diseases and HIV. Annual Reports. Vilnius. 2016 |  |
| Serbia | Unknown | Unknown | Unknown | Serbia | *Taenia spp.* | Lalosevic et al., 2012 | [3] |
| Serbia | Unknown | Unknown | Unknown | Serbia | *Taenia spp.* | Lalosevic et al., 2012 | [3] |
| Serbia | Unknown | Unknown | Unknown | Serbia | *Taenia spp.* | Lalosevic et al., 2012 | [3] |
| Serbia | Unknown | Unknown | Unknown | Serbia | *Taenia spp.* | Lalosevic et al., 2012 | [3] |
| Serbia | Unknown | Unknown | Unknown | Serbia | *T. saginata* | Lalosevic et al., 2012 | [3] |
| Serbia | Unknown | Unknown | Female | France | *Taenia spp.* | Lalosevic et al., 2012 | [3] |
| Serbia | Unknown | Unknown | Male | Serbia | *Taenia spp.* | Lalosevic et al., 2012 | [3] |

**Additional file 5:** **Table S4.** Aggregated taeniosis cases reported in documents (authorities’ reports, epidemiological bulletins, national registries and publications) identified in eastern Europe available from 1990 to 2017.

| **Country of diagnosis/report** | **Year** | **Total number of cases** | | | **Level of data collection** | **Data source** | **Other information** | **First author and year or specification** | **Reference** |
| --- | --- | --- | --- | --- | --- | --- | --- | --- | --- |
|  |  | ***Taenia* spp*.*** | ***T. saginata*** | ***T. solium*** |  |  |  |  |  |
| Albania | 1982-2002 |  |  | 18 | National | Publication |  | Zammarchi et al., 2011 | [4] |
| Bulgaria | 1982-2002 |  |  | 0 | National | Publication |  | Kurdova 2001 | [5] |
|  | 1991-2000 |  | 74 |  |  |  |  |  |  |
| Croatia | 2002 |  | 4 |  | National | Hospital records |  | University Hospital for Infectious Diseases „Dr. Fran Mihaljević“, 2016 |  |
|  | 2003 |  | 2 |  |  |  |  |  |  |
|  | 2004 |  | 3 |  |  |  |  |  |  |
|  | 2005 |  | 4 |  |  |  |  |  |  |
|  | 2006 |  | 1 |  |  |  |  |  |  |
|  | 2007 |  | 0 |  |  |  |  |  |  |
|  | 2008 |  | 4 |  |  |  |  |  |  |
|  | 2009 |  | 1 |  |  |  |  |  |  |
|  | 2010 |  | 3 |  |  |  |  |  |  |
|  | 2011 |  | 1 |  |  |  |  |  |  |
|  | 2012 |  | 0 |  |  |  |  |  |  |
|  | 2013 |  | 4 |  |  |  |  |  |  |
|  | 2014 |  | 2 |  |  |  |  |  |  |
|  | 2015 |  | 0 |  |  |  |  |  |  |
|  | 2016 |  | 0 |  |  |  |  |  |  |
|  | 1997 |  | 43 |  | National | Report | 2 imported cases | National Institute of Public Health, 2016 |  |
|  | 1998 |  | 65 |  |  |  | 15 imported cases |  |  |
|  | 1999 |  | 76 |  |  |  | 39 imported cases |  |  |
|  | 2000 |  | 65 |  |  |  | 27 imported cases |  |  |
|  | 2001 |  | 28 |  |  |  | 2 imported cases |  |  |
|  | 2002 |  | 27 |  |  |  | 4 imported cases |  |  |
|  | 2003 |  | 19 |  |  |  | 3 imported cases |  |  |
|  | 2004 |  | 20 |  |  |  | 2 imported cases |  |  |
|  | 2005 |  | 11 |  |  |  |  |  |  |
|  | 2006 |  | 13 |  |  |  | 2 imported cases |  |  |
|  | 2007 |  | 26 |  |  |  |  |  |  |
|  | 2008 |  | 7 |  |  |  |  |  |  |
|  | 2009 |  | 3 |  |  |  |  |  |  |
|  | 2010 |  | 4 |  |  |  | 3 imported cases |  |  |
|  | 2011 |  | 9 |  |  |  | 1 imported case |  |  |
|  | 2012 |  | 6 |  |  |  |  |  |  |
|  | 2013 |  | 26 |  |  |  |  |  |  |
| Estonia | 1980 |  | 4 | 1 | National | Reports of Health Board in Estonia |  | Jõgiste et al., 2000 |  |
|  | 1981 |  | 5 |  |  |  |  |  |  |
|  | 1982 |  | 2 | 1 |  |  | Age^[[1]](#footnote-1)^: ≤60, female (1 sol^[[2]](#footnote-2)^) Age: 20-29 (1 sag^[[3]](#footnote-3)^); 30-39 (1 sag) |  |  |
|  | 1983 |  | 2 | 2 |  |  | Age:40-49, female (2 sol) |  |  |
|  | 1984 |  | 3 | 2 |  |  |  |  |  |
|  | 1985 |  | 1 | 3 |  |  |  |  |  |
|  | 1986 |  | 2 | 1 |  |  | Age:40-49, male (1 sol); Age: 20-29; 30-39 (2 sag) |  |  |
|  | 1987 |  | 4 |  |  |  | Age: 20-29; 40-49; 50-59; unknown |  |  |
|  | 1988 |  | 1 |  |  |  |  |  |  |
|  | 1989 |  | 2 |  |  |  | Age: 20-29 (1); unknown (1) |  |  |
|  | 1990 |  | 3 |  |  |  | Age: 20-29 (1); 30-39 (2) |  |  |
|  | 1991 |  | 2 |  |  |  | Age: 10-19 (1); 20-29 (1) |  |  |
|  | 1992 |  | 5 |  |  |  |  |  |  |
|  | 1993 |  | 0 | 2 |  |  | Age: 5-9 (1); 60-64 (1) |  |  |
|  | 1994 |  | 2 |  |  |  | Age: 40-49 (2) |  |  |
|  | 1995 |  | 1 |  |  |  | Age: 40-49 (1) |  |  |
|  | 1996 |  | 2 |  |  |  | Age: 5-9 (1); 40-49 (1) |  |  |
|  | 1997 |  | 3 | 1 |  |  | Age: 5-9 (1 sol) Age: 0-4 (2 sag); 30-39 (1sag) |  |  |
|  | 1998 |  | 1 | 2 |  |  | Age: 5-9 (1 sol), 40-49 (1 sol) Age: 5-9 and male (1 sag) |  |  |
| Estonia | 1999 |  | 1 |  |  | Publication | Age: 40-49 and male | Jõgiste et al., 2005 |  |
|  | 2000 |  |  | 2 |  |  | Age ≥60and female and male, urban inhabitants |  |  |
| Estonia | 2003 |  |  | 1 | National | Personal communication;  Specialist of Health Board in Estonia | Age: 15-19 and female | Department of Communicable Disease Surveillance and Control, Health Board |  |
|  | 2012 |  | 1 |  |  |  | Age: >60 and female |  |  |
| Estonia | 2004–2011 |  |  | 7 | National | Publication | Serology, ELISA, among general population, age 18+ years | Lassen et al., 2016 | [6] |
|  | 2003 |  |  | 0 |  |  | Serology, ELISA, among general population, age 14-17 years |  |  |
|  | 2012 |  |  | 1 |  |  | Serology, ELISA, among veterinarians |  |  |
|  | 2013-2014 |  |  | 3 |  |  | Serology, ELISA, among animal caretakers |  |  |
|  | 2013 |  |  | 2 |  |  | Serology, ELISA, among hunters |  |  |
| Former Yugoslav Republic of Macedonia | 2006-2013 | 6 |  |  |  | Data presented at the 5th congress of Macedonian microbioloogists | Bitola, municipality Kichevo | Adamovska E 2014, Center for Public Health |  |
| Former Yugoslav Republic of Macedonia | 2005-2015 | 1 |  |  |  | Laboratory records | Prilep | Miloskoska Blagoeska E, Center for Public Health |  |
| Former Yugoslav Republic of Macedonia | 2003-2013 | 3 |  |  |  | Laboratory records | Tetovo | Jusufi F, Center for Public Health |  |
| Former Yugoslav Republic of Macedonia | 2010-2015 | 1 |  |  |  | Laboratory records | Municipality Skopje | Zaturoska D, Military Hospital in Skopje |  |
| Former Yugoslav Republic of Macedonia | 2010-2015 |  | 1 |  |  | Laboratory records | Municipality Skopje | Cvetkovic S, Institute of Public health of the Republic of Macedonia |  |
| Former Yugoslav Republic of Macedonia | 2010-2015 | 4 | 4 |  |  | Laboratory records | Municipality Skopje | Petrovska M, Jurhar Pavlova M. Institute of Microbiology and Parasitology, Medical faculty, University “Ss Cyril and Methodius”, Skopje |  |
| Hungary | 2000 | 6 |  |  | National | Poster |  | Kucsera et al., 2015 |  |
|  | 2001 | 4 |  |  |  |  |  |  |  |
|  | 2002 | 2 |  |  |  |  |  |  |  |
|  | 2003 | 3 |  |  |  |  |  |  |  |
|  | 2004 | 2 |  |  |  |  |  |  |  |
|  | 2005 | 1 |  |  |  |  |  |  |  |
|  | 2006 | 2 |  |  |  |  |  |  |  |
|  | 2008 | 3 |  |  |  |  |  |  |  |
|  | 2009 | 5 |  |  |  |  |  |  |  |
|  | 2010 | 3 |  |  |  |  |  |  |  |
|  | 2011 | 3 |  |  |  |  |  |  |  |
|  | 2012 | 7 |  |  |  |  |  |  |  |
|  | 2013 | 1 |  |  |  |  |  |  |  |
|  | 2014 | 6 |  |  |  |  |  |  |  |
| Latvia | 2000 |  |  | 3 |  | Report | Age: 18-29; 30-39; >60 | Disease Prevention and Control Centre, 2014 |  |
|  | 2001 |  |  | 1 |  |  | Age: 18-29 |  |  |
|  | 2003 |  |  | 1 |  |  | Age: 18-29 |  |  |
|  | 2005 |  |  | 1 |  |  | Age: >60 |  |  |
|  | 2006 |  |  | 1 |  |  | Age: 40-49 |  |  |
|  | 2007 |  |  | 1 |  |  | Age: >60 |  |  |
|  | 2008 |  |  | 1 |  |  | Age: 30 - 39 |  |  |
|  | 2009 |  |  | 1 |  |  | Age: 50 -59 |  |  |
|  | 2013 |  |  | 1 |  |  | Age: >60 |  |  |
| Poland | 1990 | 86 | 1193 | 7 | National | Publicationsbasing on Reports of National Institute of Hygiene |  | Nasilowska, 1992 | [7] |
|  | 1992 | 76 | 1059 | 12 |  |  |  | Plonka, 1994 | [8] |
|  | 1993 | 114 | 1361 | 4 |  |  |  | Plonka, 1995 | [9] |
|  | 1994 | 96 | 1023 | 10 |  |  |  | Plonka, 1997 | [10] |
|  | 1995 | 89 | 964 | 6 |  |  |  | Plonka, 1997 | [10] |
|  | 1996 | 57 | 730 | 6 |  |  |  | Plonka, 1998 | [11] |
|  | 1997 | 63 | 634 | 6 |  |  |  | Plonka, 1999 | [12] |
|  | 1998 | 59 | 516 | 5 |  |  |  | Plonka, 2000 | [13] |
|  | 1999 | 35 | 369 | 7 |  |  |  | Plonka, 2001 | [14] |
|  | 2000 | 52 | 359 | 3 |  |  |  | Plonka and Waloch, 2002 | [15] |
|  | 2001 | 91 | 195 | 2 |  |  |  | Waloch, 2003 | [16] |
|  | 2002 | 53 | 193 | 3 |  |  |  | Waloch, 2004 | [17] |
|  | 2003 | 32 | 157 | 4 |  |  |  | Waloch, 2005 | [18] |
|  | 2004 | 70 | 119 | 4 |  |  |  | Waloch, 2006 | [19] |
|  | 2005 | 59 | 109 | 3 |  |  |  | Waloch, 2007 | [20] |
|  | 2006 | 39 | 97 | 4 |  |  |  | Waloch, 2008 | [21] |
|  | 2007 | 30 | 84 | 4 |  |  |  | Waloch, 2009 | [22] |
|  | 2008 | 30 | 69 | 2 |  |  |  | Waloch, 2010 | [23] |
|  | 2009 | 4 | 20 | 0 |  |  |  | Waloch, 2011 | [24] |
| Republic of Cyprus |  |  | 0 | 0 |  |  |  | Economides, 2000 | [25] |
| Romania | 2007 | 412 |  |  | National |  |  | Official data provided by the  National Centre for statistics and IT in Public Health – hospitalized cases |  |
|  | 2008 | 467 |  |  |  |  |  |  |  |
|  | 2009 | 584 |  |  |  |  |  |  |  |
|  | 2010 | 457 |  |  |  |  |  |  |  |
|  | 2011 | 310 |  |  |  |  |  |  |  |
|  | 2012 | 306 |  |  |  |  |  |  |  |
|  | 2013 | 323 |  |  |  |  |  |  |  |
|  | 2014 | 306 |  |  |  |  |  |  |  |
| Serbia | 1997 | 13 |  |  | National | Reports |  | Institute of Public Health of Serbia “Dr Milan Jovanović Batut”, 1997-2004 |  |
|  | 1998 | 30 |  |  |  |  |  |  |  |
|  | 1999 | 25 |  |  |  |  |  |  |  |
|  | 2000 | 69 |  |  |  |  |  |  |  |
|  | 2001 | 47 |  |  |  |  |  |  |  |
|  | 2002 | 5 |  |  |  |  |  |  |  |
|  | 2003 | 12 |  |  |  |  |  |  |  |
|  | 2004 | 11 |  |  |  |  |  |  |  |
| Serbia | 1980-1989 | 14 | 7 |  | Reports of the Clinical Centre of Serbia Infectious Diseases Clinic | CYSTINET meeting Belgrade |  | Dakic Z et al., 2015 |  |
|  | 1990-1999 | 6 | 6 |  |  |  |  |  |  |
|  | 2000-2009 | 1 | 3 |  |  |  |  |  |  |
|  | 2010-2014 | 2 | 1 |  |  |  |  |  |  |
|  | 2015- 2017 | 0 | 2 |  |  |  |  |  |  |
| Slovakia | 2004-2014 | 10 | 20 |  |  | Report |  | Public Health Authority of the Slovak Republic |  |

**Additional file 5: Table S5.** Taeniosis prevalence data reported in epidemiological studies published between 1990-2017.

| **Country of diagnosis/report** | **Year(s)** | **Prevalence (%) or range when available** | ***Taenia* species** | **Level of data collection** | **Data source** | **Other information** | **First author and year or specification** | **Reference** |
| --- | --- | --- | --- | --- | --- | --- | --- | --- |
| Estonia | 2004–2011 | 0.7 (0.3–1.4) | *T. solium* | National | Publication | Serology, ELISA, among general population, age 18+ years | Lassen et al., 2016 | [6] |
|  | 2003 | 0.0 (0.0–1.2) |  |  |  | Serology, ELISA, among general population, age 14-17 years |  |  |
|  | 2012 | 0.6 (0.0–3.1) |  |  |  | Serology, ELISA, among veterinarians |  |  |
|  | 2013-2014 | 0.8 (0.2–2.2) |  |  |  | Serology, ELISA, among animal caretakers |  |  |
|  | 2013 | 1.4 (0.2–4.5) |  |  |  | Serology, ELISA, among hunters |  |  |
| Former Yugoslav Republic of Macedonia | before 1990 | 0.39 - 2.06 | *T. saginata* | National | Publication |  | Cuperlovic 1991 | [26] |
| Hungary | 2000-2014 | 0.01 | *T. saginata* | National | Publication |  | Kucsera et al., 2015 |  |
| Poland | 1989 | 0.66 | *T. saginata* | Regional – Katowice district | Publication |  | Derylo & Szilman 1995 | [27] |
|  | 1990 | 0.52 |  |  |  |  |  |  |
|  | 1991 | 1.28 |  |  |  |  |  |  |
|  | 1992 | 1.30 |  |  |  |  |  |  |
|  | 1993 | 1.18 |  |  |  |  |  |  |
|  | 1989 | 0.10 | *Taenia spp.* |  |  |  |  |  |
|  | 1990 | 0.18 |  |  |  |  |  |  |
|  | 1991 | 0.16 |  |  |  |  |  |  |
|  | 1992 | 0.11 |  |  |  |  |  |  |
|  | 1993 | 0.33 |  |  |  |  |  |  |
| Slovakia | NA | 4.9 | *Taenia spp.* | Regional - Roma settlements of Košice and Prešov regions | Publication | Children below 14 years | Rudohradska et al., 2012 | [28] |

**Additional file 5:** **Table S6.** Individual human cysticercosis cases identified in case reports in eastern Europe (1990-2017).

| **Country of diagnosis/report** | **Year(s)** | **Country of origin** | **Age** | **Sex** | **Travel/immigration history** | **Data source** | **First author and year or specification** | **Reference** |
| --- | --- | --- | --- | --- | --- | --- | --- | --- |
| Australia | NA | Cyprus | 54 | Female |  | Publication | Yong and Warren, 1994 | [29] |
|  | 1994 | Greece | 40 | Male | Migrated to Australia from Greece in 1957 at the age of 11; during 1977 he returned to Greece for an eleven weeks holiday to urban and rural areas in Greece, where a local diet was consumed. He initially presented in 1982 to The Sydney Hospital, Austalia |  | Davies et al., 1996 | [30] |
|  | NA | Czechoslovakia | 64 | Male | Czechoslovakian origin, had become seriously ill while visiting his home land 6 years previously |  | Gubbay et al., 1998 | [31] |
| Austria | 1996 | Serbia | 48 | Unknown | Serbian origin | Publication | Finsterer et al., 2001 | [32] |
| Croatia | 1988 | Unknown | 72 | Female | The majority of patients were from rural regions, but migration of individuals during the studied period revealed that involved aggression toward and war in Croatia should be taken into consideration | Publication | Talan-Hranilovic et al., 2002 | [33] |
|  | 1989 | Unknown | 49 | Male |  |  |  |  |
|  | 1994 | Unknown | 42 | Female |  |  |  |  |
|  | 1995 | Unknown | 77 | Male |  |  |  |  |
|  | 1995 | Unknown | 40 | Female |  |  |  |  |
|  | 1996 | Unknown | 52 | Female |  |  |  |  |
|  | 2000 | Unknown | 67 | Male |  |  |  |  |
|  | NA | Unknown | 70 | Male | Unknown |  | Titlic et al., 2007 | [34] |
|  | NA | Croatia | 59 | Unknown | Unknown |  | Gašparić et al., 2004 |  |
| Czech Republic | 1971 | Czech Republic | 50 | Male | No known travel history | Publication | Vanista et al., 1993 | [35] |
|  | 1972 | Czech Republic | 47 | Male | No known travel history |  |  |  |
|  | 1972 | Czech Republic | 40 | Female | Travelled to Korea |  |  |  |
|  | 1978 | Czech Republic | 68 | Female | Travelled to Ukraine |  |  |  |
|  | 1981 | Czech Republic | 34 | Female | Travelled to India |  |  |  |
|  | 1984 | Laos | 36 | Male | Travelled to Laos |  |  |  |
|  | 1985 | Namibia | 36 | Male | Travelled to Namibia, Angola |  |  |  |
|  | 1985 | Czech Republic | 30 | Female | Travelled to Vietnam, Cuba |  |  |  |
|  | 1990 | Zimbabwe | 25 | Male | Travelled to Zimbabwe |  |  |  |
|  | 1990 | Vietnam | 21 | Male | Travelled to/from Vietnam |  |  |  |
| Czechoslovakia | NA | Unknown | Unknown | Unknown | Unknown | Publication | Valkounova et al., 1992 | [36] |
| Denmark | NA | Yugoslavia | 20 | Female | Yugoslavian origin | Publication | Juhl and Logager, 2000 | [37] |
| Greece | NA | Unknown | 43 | Male | Unknown | Publication | Palasis and Drevelengas, 1991 | [38] |
|  | NA | Unknown | 60 | Male | Farmer, otherwise healthy | Publication | Paterakis et al., 2007 also reported by Unit Director Georgakoulas (General Hospital of Athens -G. Gennimatas) | [39] |
|  | 1997 | Germany | Unknown | Male | Permanent resident of Santorini island | personal comm. | Dr Toulios, 2016 |  |
|  | 20 years ago | NA | Unknown | Unknown | Case referred at George Papanikolaou General Hospital of Thessaloniki |  | Dr Bostantzopoulou, 2016 |  |
|  | 25 years ago | VelventoKozani, Greece | 53 |  | Case referred at AHEPA Hospital of Thessaloniki |  | Dr Bostantzopoulou, 2016 |  |
|  | 2000 | India | 29 | Male | Cased referred at Venizeleio – Pananeio General Hospital of Irakleion |  | Dr Vlachos, 2016 |  |
|  | 2009 | Unknown | 70 | Male | General University Hospital of Patras | Publication |  |  |
|  | 2004 | Unknown | 69 | Male | History of travelling abroad | Publication |  |  |
|  | NA | Unknown | 47 | Female | Frequent trips to Brazil 15 years before she was diagnosed with neurocysticercosis | Publication | Nanassis et al., 1993 |  |
|  | NA | Unknown | 47 | Male | Unknown | Publication | Nanassis et al., 1993 |  |
|  | NA | Unknown | 56 | Female | Travel history to Asia | Publication | Neroutsos et al., 2008 |  |
| Hungary |  | Unknown | 46 | Male | Unknown | Publication | Meszaros et al., 2003 | [40] |
|  |  | Unknown | 29 | Female | Unknown |  |  |  |
| Latvia | NA | Unknown | 42 | Female | Visited an endemic region | Publication | Aksiks and Sverzickis, 2007 | [41] |
|  | NA | Unknown | 49 | Female | Contact with raw pork and other people handling it |  |  |  |
| Lithuania | 2006 | Unknown | 45-54 | Male | Unknown | Report | Lithuanian Centre for Infectious Diseases and HIV. Annual Reports. Vilnius. 2006 |  |
| Lithuania | 2008 | Unknown | 45-55 | Male | Unknown | Report | Lithuanian Centre for Infectious Diseases and HIV. Annual Reports. Vilnius. 2008 |  |
| Lithuania | 2012 | Unknown | 25-34 | Male | Unknown | Report | Lithuanian Centre for Infectious Diseases and HIV. Annual Reports. Vilnius. 2012 |  |
| Lithuania | 2016 | Unknown | 55-64 | Male | Unknown | Report | Lithuanian Centre for Infectious Diseases and HIV. Annual Reports. Vilnius. 2016 |  |
| Lithuania | 2016 | Unknown | 55-65 | Male | Unknown | Report |  |  |
| Serbia | 1994 | Serbia | 49 | Male | Unknown | Publication | Nozić et al, 1995 |  |
|  | 1995 | Bosnia and Herzegovina | 84 | Female | Unknown |  | Babić et al, 1996 |  |
|  |  | Serbia | Unknown | Male | Unknown |  | Aksentijević et al. 2005 |  |
|  | NA | Serbia | 79 | Male | Unknown |  | Potić et al. 2013 |  |
|  | 2008 | Unknown | 30 | Female | Unknown |  | Vasiljevic-Vuckovic et al., 2011 | [42] |
| Slovenia | 2001 | Former Yugoslav Republic | 51 | Male | Unknown | Publication | Soba et al., 2014 | [43] |
|  | 2001 | Bosnia and Herzegovina | 41 | Female | Unknown |  |  |  |
|  | 2003 | Former Yugoslav Republic | 41 | Male | Unknown |  |  |  |
|  | 2004 | Bosnia and Herzegovina | 47 | Female | Unknown |  |  |  |
|  | 2006 | Bosnia and Herzegovina | 40 | Female | Unknown |  |  |  |
|  | 2010 | Bosnia and Herzegovina | 58 | Female | Unknown |  |  |  |
|  | 2012 | Serbia | 31 | Male | Was believed to have acquired the infection while a shepherd in Bosnia and Herzegovina |  |  |  |
| Switzerland | Unknown | Croatia | 49 | Female | Croatian origin, but lived for 22 years in Switzerland | Publication | Bauer et al., 1994 | [44] |

**Additional file 5:** **Table S7.** Aggregated human cysticercosis cases identified in case reports and publications in eastern Europe (1990-2017).

| **Country** | **Year** | **No. of cases** | **Data source** | **Other info** | **First author and Year or unpublished source** | **Reference** |
| --- | --- | --- | --- | --- | --- | --- |
| Bosnia and Herzegovina | 2001-2010 | 4 |  |  | Soba et al., 2014 | [43] |
| Bulgaria | 1991-2000 | 1-3 each year |  | Unknown | Kurdova, 2001 | [5] |
| Croatia | 2002 | 5/119 |  | Data based on serology | Department of Parasitology and Parasitic Diseases with Clinic, Faculty of Veterinary Medicine of University of Zagreb |  |
|  | 2003 | 5/57 |  |  |  |  |
|  | 2004 | 0/55 |  |  |  |  |
|  | 2005 | 4/72 |  |  |  |  |
|  | 2006 | 1/62 |  |  |  |  |
|  | 2007 | 1/61 |  |  |  |  |
|  | 2008 | 0/73 |  |  |  |  |
|  | 2009 | 1/26 |  |  |  |  |
|  | 2010 | 3/59 |  |  |  |  |
|  | 2011 | 1/42 |  |  |  |  |
|  | 2012 | 1/146 |  |  |  |  |
|  | 2013 | 0/9 |  |  |  |  |
|  | 2014 | 0/16 |  |  |  |  |
|  | 2015 | 0/8 |  |  |  |  |
|  | 2016 | 0/9 |  |  |  |  |
| Croatia | 2005-2009 | 11/770 |  | Cysticercosis Western Blot (WB) IgG test (LDBIO diagnostics, Lyon, France), people with epilepsy | Meštrović T et al., 2012 | [45] |
| Czech Republic | N/A | 30-40 each year |  | Unknown | Kolářová, 2006 | [46] |
| Former Yugoslav Republic of Macedonia | 1994-2008 | 5 | Laboratory report | mean age 34.5 years (22-44 years), 3 were female and 2 male, histopathology examination on 4873 material after neurosurgical interventions. | Ilievski, B., Petrusevska G, 2016 |  |
|  | 2008-2015 | 0 | Laboratory report | Histopathology examination on 4873 material after neurosurgical interventions. |  |  |
| Former Yugoslav Republic of Macedonia | 2014_2015 | 2 | Hospital record (among people with epilepsy) | N/A  CT and MRI | Kuzmanovski I, 2016 |  |
| Greece | 1987-1992 | 4 |  | 2 female and 2 male | Nanassis et al., 1999 |  |
|  | N/A | 4 |  | 4 cases of cysticercosis managed in Venizeleio – Pananeio General Hospital of Irakleion Crete, |  |  |
| Kosovo | N/A | 14 |  | 14 cysticercosis cases managed in Pristina General Hospital in Kosovo | Nanassis et al., 1999 |  |
| Latvia | 2003 | 1 |  | Age: 30-39 | Disease Prevention and Control Centre, 2014 |  |
|  | 2004 | 1 |  | Age: 50-59 |  |  |
|  | 2005 | 2 |  | Age: >60 |  |  |
|  | 2006 | 2 |  | Age: 30-39; >60 |  |  |
|  | 2007 | 1 |  | Age: 50-59 |  |  |
|  | 2010 | 1 |  | Age: 40-49 |  |  |
| Poland | 2010 | 2 | Publication basing on Reports of National Institute of Hygiene | Unknown | Czarkowski and Gołąb, 2013 | [47] |
|  | 2011 | 2 | Publication basing on Reports of National Institute of Hygiene | Age: 33 male; 22 female | Czarkowski and Gołąb, 2013 | [47] |
|  | 2012 | 0 |  | Unknown | Gołąb et al., 2014 | [48] |
| Poland | 1996 | 0 | Reports of National Institute of Hygiene |  | Annual Report of infectious diseases and poisonings in Poland, 1997 |  |
|  | 1997 | 4 | Publication basing on Reports of National Institute of Hygiene | two females living in a town in Łodzkie province and one male and one female living in a city in Dolnośląskie province | Płonka , 1999 | [12] |
|  | 1998 | 3 |  | two males one living in a village and one in a town in Łodzkie province and one male living in a village in Mazowieckie province | Płonka, 2000 | [13] |
|  | 1999 | 0 |  |  | Plonka, 2001 | [14] |
|  | 2000 | 0 |  |  | Plonka and Waloch, 2002 | [15] |
|  | 2001 | 0 |  |  | Waloch, 2003 | [16] |
|  | 2002 | 0 |  |  | Waloch, 2004 | [17] |
|  | 2003 | 0 |  |  | Waloch, 2005 | [18] |
|  | 2004 | 0 |  |  | Waloch, 2006 | [19] |
|  | 2005 | 0 |  |  | Waloch, 2007 | [20] |
|  | 2006 | 1 |  | 1 person from Kujawsko-pomorskie province | Waloch, 2008 | [21] |
|  | 2007 | 0 |  |  | Waloch, 2009 | [22] |
|  | 2008 | 1 |  | 1 person from Podlaskie province | Waloch, 2010 | [23] |
|  | 2009 | 0 |  |  | Waloch, 2011 | [24] |
|  | 2010 | 0 |  |  | Waloch, 2012 | [49] |
|  | 2011 | 2 | Reports of National Institute of Hygiene | One male 33 years old living in a town in Małopolskie province and one female 24 years old living in a village in Mazowieckie province | Reports of National Institute of Hygiene, 2013 |  |
|  | 2012 | 0 |  |  | Reports of National Institute of Hygiene, 2013 |  |
|  | 2013 | 3 |  |  | Czarkowski et al., 2014 |  |
| Romania | 2008 | 44 |  | Cysticercosis central nervous system | Official data provided by the  National Centre for statistics and IT in Public Health – hospitalized cases |  |
|  |  | 4 |  | Cysticercosis unknown |  |  |
|  | 2009 | 37 |  | Cysticercosis central nervous system |  |  |
|  |  | 1 |  | Cysticercosis in the eye |  |  |
|  |  | 1 |  | Cysticercosis other location |  |  |
|  |  | 3 |  | Cysticercosis unknown |  |  |
|  | 2010 | 52 |  | Cysticercosis central nervous system |  |  |
|  |  | 5 |  | Cysticercosis other location |  |  |
|  |  | 1 |  | Cysticercosis unknown |  |  |
|  | 2011 | 52 |  | Cysticercosis central nervous system |  |  |
|  |  | 8 |  | Cysticercosis other location |  |  |
|  |  | 1 |  | Cysticercosis unknown |  |  |
|  | 2012 | 48 |  | Cysticercosis central nervous system |  |  |
|  |  | 1 |  | Cysticercosis in the eye |  |  |
|  |  | 1 |  | Cysticercosis unknown |  |  |
|  | 2013 | 61 |  | Cysticercosis central nervous system |  |  |
|  | 2014 | 54 |  | Cysticercosis central nervous system |  |  |
| Serbia | 1990-1994 | 24 | Reports on Infectious Diseases Clinics in Belgrade and Novi Sad | All cases of neurocysticercosis from Serbia between 1990 and 2014:  Age between 7 and 73 years, with a mean of 46.2 ± 1.1. | Bobić et al, 2015 |  |
|  | 1995-1999 | 77 |  |  |  |  |
|  | 2000-2004 | 50 |  |  |  |  |
|  | 2005-2009 | 16 |  |  |  |  |
|  | 2010-2014 | 1 |  |  |  |  |
|  | 1997-2001 | 13 | Reports on Infectious Diseases Clinics in Novi Sad | North Serbia; age between 35 and 63 years, mean of 49 | Doder et al, 2002 | [50] |
|  | 2000-2004 | 60 | Reports on Infectious Diseases Clinics in Belgrade | All patients treated at the clinic in the specified period | Nikolić, Stevanović 2006 | [51] |
|  | 2006-2010 | 22 | Reports on Infectious Diseases Clinics in Belgrade | 19 patients from Serbia, 3 patients from, Bosnia and Herzegovina | Poluga et al 2013 |  |
|  | 2005-2006 | 12 |  | 4 men, 8 women, mean age 56,4 years | Milovanovic et al., 2008 | [52] |

**Additional file 5:** **Table S8.** Porcine cysticercosis cases identified and reported during meat inspection in case reports and publications in eastern Europe (1990-2017).

| **Country** | **Prevalence** | **Cases/sampled** | **Timeframe** | **Level of data collection** | **Reported as** | **Other info** | **First author and year or unpublished source** | **Reference** |
| --- | --- | --- | --- | --- | --- | --- | --- | --- |
| Bulgaria | 0.016 | 3/18714 | 2009 | Whole country |  |  | Devleesschauwer et al., 2017 | [53] |
|  | 0.04 | 12/29112 | 2008 |  |  |  |  |  |
|  | 0.14 | 33/23441 | 2007 |  |  |  |  |  |
|  | 0.18 | 468/264996 | 2006 |  |  |  |  |  |
|  |  | 341/NA | 2005 |  |  |  |  |  |
| Estonia | 0.00 | 0/1217 | 2014 | Three large slaugtherhouses |  | One cyst found but could not be confirmed by PCR | Dorbek-Kolin et al., 2018 | [54] |
| Estonia |  | 10/NA | 2006 |  |  | Reported by Food and Veterinary Service, Latvia | Dorny et al., 2010 | [55] |
| Former Yugoslavia | 0.008 | 274/3202772 | 1985-1989 |  |  |  | Čuperlović 1991 |  |
| Former Yugoslav Republic of Macedonia | 0 | 0 | 2006-2016 |  |  |  | Food and veterinary agency, 2016 |  |
| Lithuania | 0.01 | 113/NA | 2006 |  |  | Reported by Food and Veterinary Service, Latvia | Dorny et al., 2010 | [55] |
| Lithuania |  | 580/807256 | 1998 | Abattoir level / whole country |  |  | Lithuanian State Food and Veterinary Service, 1998 |  |
|  |  | 640/849433 | 1999 |  |  |  | Lithuanian State Food and Veterinary Service, 1999 |  |
|  |  | 222/676598 | 2000 |  |  |  | Lithuanian State Food and Veterinary Service, 2000 |  |
|  |  | 212/783768 | 2001 |  |  |  | Lithuanian State Food and Veterinary Service, 2001 |  |
|  |  | 287/938694 | 2002 |  |  |  | Lithuanian State Food and Veterinary Service, 2002 |  |
|  |  | 293/1000207 | 2003 |  |  |  | Lithuanian State Food and Veterinary Service, 2003 |  |
|  |  | 406/890145 | 2004 |  |  |  | Lithuanian State Food and Veterinary Service, 2004 |  |
|  |  | 167/971951 | 2005 |  |  |  | Lithuanian State Food and Veterinary Service, 2005 |  |
|  |  | 295/965540 | 2006 |  |  |  | Lithuanian State Food and Veterinary Service, 2006 |  |
|  |  | 113/928701 | 2007 |  |  |  | Lithuanian State Food and Veterinary Service, 2007 |  |
|  |  | 137/694827 | 2008 |  |  |  | Lithuanian State Food and Veterinary Service, 2008 |  |
|  |  | 95/551811 | 2009 |  |  |  | Lithuanian State Food and Veterinary Service, 2009 |  |
|  |  | 101/721075 | 2010 |  |  |  | Lithuanian State Food and Veterinary Service, 2010 |  |
|  |  | 43/770395 | 2011 |  |  |  | Lithuanian State Food and Veterinary Service, 2011 |  |
|  |  | 8/790667 | 2012 |  |  |  | Lithuanian State Food and Veterinary Service, 2012 |  |
|  |  | 2/878470 | 2013 |  |  |  | Lithuanian State Food and Veterinary Service, 2013 |  |
|  |  | 4/888663 | 2014 |  |  |  | Lithuanian State Food and Veterinary Service, 2014 |  |
|  |  | 8/860237 | 2015 |  |  |  | Lithuanian State Food and Veterinary Service, 2015 |  |
|  |  | 2/784134 | 2016 |  |  |  | Lithuanian State Food and Veterinary Service, 2016 |  |
|  |  | 0/790870 | 2017 |  |  |  | Lithuanian State Food and Veterinary Service, 2017 |  |
| Moldova | 0.0005 | 1/218653 | 2013 |  |  |  | National Agency for Food Safety, 2017 |  |
|  | 0.0004 | 1/261815 | 2015 |  |  |  |  |  |
|  | 0.0004 | 1/267123 | 2017 |  |  |  |  |  |
| Poland | 0.00016 | 30/18672315 | 2000 |  |  | Publication basing on reports of Veterinary Inspection Service | Lis, 2002 | [56] |
| Poland | 0.00020 | 35/16958775 | 1997 |  |  | Publication basing on reports of Veterinary Inspection Service | Lis, 1999 | [57] |
| Poland | 0.00027 | 38/14067561 | 1994 |  |  | Publication basing on reports of Veterinary Inspection Service | Lis, 1998 | [58] |
| Poland | 0.0002 | 1/567151 | 1987 | Katowice district | Cysticercus cellulosae | Publication basing on reports of Veterinary Inspection Service | Derylo and Szilman 1995 | [27] |
|  | 0.0002 | 1/629477 | 1988 | Katowice district |  |  |  |  |
|  | 0.0005 | 4/862502 | 1990 | Katowice district |  |  |  |  |
| Poland | 0.0013 | 11/839886 | 2005 | Lublin Province | Cysticercus cellulosae | Publication basing on reports of Veterinary Inspection Service | Kozlowska-Loj 2011 | [59] |
| Poland | 0.002 | 23/855938 | 2011 | Lublin Province | Cysticercus cellulosae | Publication basing on reports of Veterinary Inspection Service | Kozlowska-Loj and Loj-Maczulska, 2014 | [60] |
|  | 0.0034 | 26/756239 | 2012 |  |  |  |  |  |
| Poland | 0.0037 | 10/268225 | 2007 | Lublin Province | Cysticercus cellulosae | Publication basing on reports of Veterinary Inspection Service | Kozlowska-Loj 2011 | [59] |
| Poland | 0.004 | 37/906876 | 2010 | Lublin Province | Cysticercus cellulosae | Publication basing on reports of Veterinary Inspection Service | Kozlowska-Loj and Loj-Maczulska, 2014 | [60] |
|  | 0.007 | 64/848391 | 2009 |  |  |  |  |  |
| Poland | 0.0119 | 21/176062 | 2008 | Lublin Province | Cysticercus cellulosae | Publication basing on reports of Veterinary Inspection Service | Kozlowska-Loj 2011 | [59] |
|  | 0.0174 | 51/293197 | 2006 |  |  |  |  |  |
| Poland | 0 | 0/561241 | 1986 | Katowice district | Cysticercus cellulosae | Publication basing on reports of Veterinary Inspection Service | Derylo and Szilman 1995 | [27] |
|  | 0 | 0/765213 | 1989 | Katowice district |  |  |  |  |
|  | 0 | 0/772937 | 1991 | Katowice district |  |  |  |  |
|  | 0 | 0/710623 | 1992 | Katowice district |  |  |  |  |
|  | 0 | 0/561000 | 1993 | Katowice district |  |  |  |  |
|  | 0 | 0/500817 | 1994 | Katowice district |  |  |  |  |
| Poland |  | 4287 | 2003 |  | Report | Cysticercosis in pigs reported by Veterinary inspection Services during postmortem examination in slaughterhouses. NB: species not specified cysticercosis could refer to Cysticercus cellulosae or other species like C. tenuicollis | Veterinary inspection Services Annual Reports (2003-2013) |  |
|  |  | 19 | 2004 |  |  |  |  |  |
|  |  | 1878 | 2005 |  |  |  |  |  |
|  |  | 257 | 2006 |  |  |  |  |  |
|  |  | 3732 | 2007 |  |  |  |  |  |
|  |  | 25 | 2008 |  |  |  |  |  |
|  |  | 145 | 2009 |  |  |  |  |  |
|  |  | 849 | 2010 |  |  |  |  |  |
|  |  | 28 | 2011 |  |  |  |  |  |
|  |  | 34 | 2012 |  |  |  |  |  |
|  |  | 46 | 2013 |  |  |  |  |  |
| Republic of Cyprus | 0 |  | 1970-1999 |  |  |  | Ministry of Agriculture, Natural Resources and Environment, Nicosia, Cyprus, 1970-1999 |  |
| Romania | 9.4 | 3 out of 32 | 2013 | Ialomita Region |  |  | Devleesschauwer et al., 2017 | [53] |
| Romania | 0.001 | 89204 | 2013 |  |  | four counties: Bihor, Bistrița-Năsăud, Satu-Mare and Cluj | Oleleu et al., 2016 | [61] |
| Romania |  | 2 | 2011 | Satu Mare Region |  |  | Devleesschauwer et al., 2017 | [53] |
| Romania |  | 2 out of 3 | 2015 | Ialomita Region |  |  | <http://www.oie.int/wahis_2/public/wahid.php/Diseaseinformation/statusdetail>, Accessed 2017 |  |
|  |  | 1 out of 1 | 2015 | Arges Region |  |  |  |  |
|  |  | 6 out of 50 | 2016 | Ialomita Region |  |  |  |  |
| Romania |  | 50 | 2007 |  |  | Reported by the Faculty of Veterinary Medicine of Cluj Napoca | Dorny et al., 2010 | [55] |
| Serbia | 0.007 | 1/15085 | 1998 |  |  | Pigs slaughtered at Slaughterhouse "Mesokombinat" in Jablanica district, Republic of Serbia | Popović 2002 |  |
|  | 0.008 | 1/12300 | 1994 |  |  |  |  |  |
|  | 0.008 | 1/12164 | 1999 |  |  |  |  |  |
|  | 0.018 | 2/11213 | 1997 |  |  |  |  |  |
|  | 0.021 | 2/9379 | 1996 |  |  |  |  |  |
|  | 0.024 | 2/8172 | 1995 |  |  |  |  |  |
|  | 0.027 | 2/7510 | 2000 |  |  |  |  |  |
|  |  | 1 | 2003 |  |  |  | Ministry of Agriculture and Environmental Protection http://www.vet.minpolj.gov.rs/srb/epizootiologija/zarazne-bolesti-zivotinja |  |
|  |  | 4 | 2006 |  |  |  |  |  |
| Serbia |  | 130 | 2009 |  |  |  |  |  |
| Serbia |  | 65 | 2009 | Whole country |  |  | Devleesschauwer et al., 2017 | [53] |
| Serbia & Montenegro |  | 4 | 2006 |  |  |  |  |  |
| Slovakia | 0.002-0.006 |  | 2000-2008 |  |  |  | State Veterinary and Food Administration of the Slovak Republic, 2016 |  |
| Slovakia | 0.03 | 2out of 60 | 2014 | Banska Bystrica Region |  |  | Devleesschauwer et al., 2017 | [53] |
|  | 0.005 | 1/180 | 2013 |  |  |  |  |  |

**Additional file 5:** **Table S9.** Bovine cysticercosis cases identified and reported during meat inspection in case reports and publications in eastern Europe (1990-2017).

| **Country** | **Prevalence %** | **Cases/sampled** | **Timeframe** | **Level of data collection** | **Other info** | **First author and Year or unpublished source** | **Reference** |
| --- | --- | --- | --- | --- | --- | --- | --- |
| Belarus |  | 39 | 2005 | Abattoir level / Minsk and Mogilev region | OIE database | <http://www.oie.int/wahis_2/public/wahid.php/Diseaseinformation/statusdetail>, Accessed 2017 |  |
| Bosnia and Herzigovina | 2.42 | 405/16736 | 1965-1991 | abattoir level | Cattle slaughtered at Slaughterhouse "Pomurka" in Murska Sobota, Republic of Slovenia | Zivkovic et al., 1996 | [62] |
| Bulgaria |  | 5217 | 2005 | abattoir level / whole country | OIE database | <http://www.oie.int/wahis_2/public/wahid.php/Diseaseinformation/statusdetail>, Accessed 2017 |  |
| Croatia | 0.37 | 75/20056 | 2005 |  | Publication | Zdolec et al., 2012 | [63] |
|  | 0.049 | 17/34694 | 2006 |  |  |  |  |
|  | 0.1 | 47/41949 | 2007 |  |  |  |  |
|  | 0.1 | 42/40112 | 2008 |  |  |  |  |
|  | 0.07 | 23/31020 | 2009 |  |  |  |  |
|  | 0.067 | 24/35382 | 2010 |  |  |  |  |
| Croatia | 0.16 | 82/51536 | 1965-1991 | abattoir level | cattle slaughtered at Slaughterhouse "Pomurka" in Murska Sobota, Republic of Slovenia | Zivkovic et al., 1996 | [62] |
| Czech Republic | 1.3 |  | 1990-1992 |  |  | Official datasets of the Czech State Veterinary Administration |  |
| Czech Republic | 0.78 |  | 1993 |  |  |  |  |
| Czech Republic | 0.17 |  | 2001 |  |  |  |  |
| Czech Republic |  | 152 | 2008 |  |  |  |  |
| Czech Republic |  | 155 | 2009 |  |  |  |  |
| Czech Republic |  | 53 | 2012 |  |  |  |  |
| Estonia |  | 10/NA | 2006 |  | Reported by a referent of "Food Safety, Animal Health and Environment" "BIOR" | Dorny et al., 2010 | [55] |
| Estonia | 0.00 | 0/564 | 2014 |  | Two cysticerci found but could not be confirmed by PCR | Dorbek-Kolin et al., 2018 | [54] |
| Former Yugoslav Republic of Macedonia | 0.00 | 0 | 2006-2016 |  | Report | Food and veterinary agency, 2016 |  |
| Greece |  | 6/208 | 2005 | Abattoir level / whole country | OIE database | <http://www.oie.int/wahis_2/public/wahid.php/Diseaseinformation/statusdetail>, Accessed 2017 |  |
| Lithuania |  | 10/NA | 2005 |  | Reported by Food and Veterinary Service, Lithuania | Dorny et al., 2010 | [55] |
| Lithuania |  | 46/367431 | 1998 | Abattoir level / whole country |  | Lithuanian State Food and Veterinary Service, 1998 |  |
|  |  | 22/336326 | 1999 |  |  | Lithuanian State Food and Veterinary Service, 1999 |  |
|  |  | 19/367135 | 2000 |  |  | Lithuanian State Food and Veterinary Service, 2000 |  |
|  |  | 21/241879 | 2001 |  |  | Lithuanian State Food and Veterinary Service, 2001 |  |
|  |  | 26/197977 | 2002 |  |  | Lithuanian State Food and Veterinary Service, 2002 |  |
|  |  | 20/258894 | 2003 |  |  | Lithuanian State Food and Veterinary Service, 2003 |  |
|  |  | 8/249049 | 2004 |  |  | Lithuanian State Food and Veterinary Service, 2004 |  |
|  |  | 0/249693 | 2005 |  |  | Lithuanian State Food and Veterinary Service, 2005 |  |
|  |  | 0/216826 | 2006 |  |  | Lithuanian State Food and Veterinary Service, 2006 |  |
|  |  | 0/246678 | 2007 |  |  | Lithuanian State Food and Veterinary Service, 2007 |  |
|  |  | 1/212217 | 2008 |  |  | Lithuanian State Food and Veterinary Service, 2008 |  |
|  |  | 0/185787 | 2009 |  |  | Lithuanian State Food and Veterinary Service, 2009 |  |
|  |  | 0/178705 | 2010 |  |  | Lithuanian State Food and Veterinary Service, 2010 |  |
|  |  | 0/170606 | 2011 |  |  | Lithuanian State Food and Veterinary Service, 2011 |  |
|  |  | 0/165381 | 2012 |  |  | Lithuanian State Food and Veterinary Service, 2012 |  |
|  |  | 0/153376 | 2013 |  |  | Lithuanian State Food and Veterinary Service, 2013 |  |
|  |  | 0/159585 | 2014 |  |  | Lithuanian State Food and Veterinary Service, 2014 |  |
|  |  | 0/177220 | 2015 |  |  | Lithuanian State Food and Veterinary Service, 2015 |  |
|  |  | 1/171243 | 2016 |  |  | Lithuanian State Food and Veterinary Service, 2016 |  |
|  |  | 1/157289 | 2017 |  |  | Lithuanian State Food and Veterinary Service, 2017 |  |
| Moldova | 0.00 | 0 | 2013 |  |  | National Agency for Food Safety |  |
|  | 0.01 | 0 | 2015 |  |  |  |  |
|  | 0.02 | 0 | 2017 |  |  |  |  |
| Poland | 0.29 | 415/141015 | 2005 | Lublin Province | Publication basing on reports of Veterinary Inspection Service | Kozlowska-Loj 2011 | [59] |
|  | 0.22 | 305/137645 | 2006 |  |  | Kozlowska-Loj 2011 |  |
|  | 1.19 | 327/27421 | 2007 |  |  | Kozlowska-Loj 2011 |  |
|  | 1.49 | 438/29355 | 2008 |  |  | Kozlowska-Loj 2011 |  |
| Poland | 0.15 | 248/157683 | 2009 | Lublin Province | Publication basing on reports of Veterinary Inspection Service | Kozlowska-Loj and Loj-Maczulska, 2014 | [60] |
|  | 0.18 | 288/157763 | 2010 |  |  |  |  |
|  | 0.23 | 282/122030 | 2011 |  |  |  |  |
|  | 0.20 | 220/105487 | 2012 |  |  |  |  |
| Poland | 0.003 | 4718/1707720 | 1994 | Nationwide | Publication basing on reports of Veterinary Inspection Service | Lis, 1997 | [64] |
| Poland | 0.002 | 3854/1590065 | 1997 | Nationwide | Publication basing on reports of Veterinary Inspection Service | Lis, 1999 | [57] |
| Poland | 0.002 | 2480/1346675 | 2000 | Nationwide | Publication basing on reports of Veterinary Inspection Service | Lis, 2002 | [56] |
| Poland | 0.24 | 328/135168 | 1986 | Katowice district | Cysticercus bovis  Publication basing on reports of Veterinary Inspection Service | Derylo and Szilman 1995 | [27] |
|  | 0.26 | 320/125377 | 1987 |  |  |  |  |
|  | 0.21 | 218/106241 | 1988 |  |  |  |  |
|  | 0.17 | 146/83548 | 1989 |  |  |  |  |
|  | 0.22 | 267/123269 | 1990 |  |  |  |  |
|  | 0.16 | 200/122223 | 1991 |  |  |  |  |
|  | 0.08 | 72/90624 | 1992 |  |  |  |  |
|  | 0.12 | 87/72572 | 1993 |  |  |  |  |
|  | 0.05 | 46/75180 | 1994 |  |  |  |  |
| Poland |  | 2281 | 2003 |  | Report NB: species not specified cysticercosis could refer to Cysticercus bovis or other species like C. tenuicollis | Veterinary inspection Services Annual Reports (2003-2013) |  |
| Poland |  | 2776 | 2004 |  |  |  |  |
| Poland |  | 1889 | 2005 |  |  |  |  |
| Poland |  | 1610 | 2006 |  |  |  |  |
| Poland |  | 2 | 2007 |  |  |  |  |
| Poland |  | 1131 | 2008 |  |  |  |  |
| Poland |  | 911 | 2009 |  |  |  |  |
| Poland |  | 783 | 2010 |  |  |  |  |
| Poland |  | 778 | 2011 |  |  |  |  |
| Poland |  | 679 | 2012 |  |  |  |  |
| Poland |  | 898 | 2013 |  |  |  |  |
| Romania |  | 12/23663 | 2009 |  | Publication | Oleleu et al., 2015 | [65] |
|  |  | 0/28077 | 2010 |  |  |  |  |
|  |  | 0/21014 | 2011 |  |  |  |  |
|  |  | 0/17951 | 2012 |  |  |  |  |
|  |  | 0/17138 | 2013 |  | Publication |  |  |
| Romania | 0.15 | 11/7159 | 2013 |  | Abstract | Oleleu et al., 2014 |  |
| Serbia | 0.79 | 5/630 | 1994 |  | Cattle slaughtered at Slaughterhouse "Mesokombinat" in Jablanica district, Republic of Serbia | Popovic 2002 |  |
|  | 0.52 | 4/765 | 1995 |  |  |  |  |
|  | 0.66 | 3/457 | 1996 |  |  |  |  |
|  | 0.51 | 3/591 | 1997 |  |  |  |  |
|  | 0.58 | 2/345 | 1998 |  |  |  |  |
|  | 0.42 | 2/478 | 1999 |  |  |  |  |
|  | 0.3 | 2/657 | 2000 |  |  |  |  |
|  | 0.62 | 219/35303 | 1998-1999 |  |  |  |  |
| Serbia | 0.16 | 14/8504 | 1965-1991 | Abattoir level | cattle slaughtered at Slaughterhouse "Pomurka" in Murska Sobota, Republic of Slovenia | Zivkovic et al., 1996 | [62] |
| Serbia |  | 1 | 2007 | Zlatibor district | Information reported to the veterinary authorities | Lalosevic et al., 2012 | [3] |
|  |  | 1 | 2010 |  |  |  |  |
|  |  | 1 | 2011 |  |  |  |  |
| Serbia |  | 9/12 | 2004 | Farm in Vojvodina | Outbreak in a farm in Vojvodina | Lalosevic et al., 2012 | [3] |
| Serbia |  | 129/416 | 2004 | Farm in Bač region, Northern Serbia | Publication | Novakov et al., 2010 | [66] |
|  | 0.626 |  | 1988-1989 | Abattoir facility MIP Požarevac |  |  |  |
| Serbia | 0.63 | 225/35946 | 1988-1989 | Abattoir facility MIP Požarevac | Publication | Aleksic and Miloradovic 1994 | [67] |
| Serbia |  | 337 | 2005 | Abattoir level / whole country | OIE database | <http://www.oie.int/wahis_2/public/wahid.php/Diseaseinformation/statusdetail>, Accessed 2017 |  |
| Slovakia | 0.13 |  | 2000 |  | Report | Ministry of Agriculture and Rural Development of the Slovak Republic |  |
|  | 0.01 |  | 2014 |  |  |  |  |
| Slovakia | 0.92 |  | 1989 | Nationwide | CYSTINET Meeting Evora (Oral presentation) | Várady & Dvorožňáková, 2014 |  |
|  | 0.8 |  | 1990 |  |  |  |  |
|  | 0.7 |  | 1991 |  |  |  |  |
|  | 0.6 |  | 1992 |  |  |  |  |
|  | 0.5 |  | 1993 |  |  |  |  |
|  | 0.47 |  | 1994 |  |  |  |  |
|  | 0.35 |  | 1995 |  |  |  |  |
|  | 0.3 |  | 1996 |  |  |  |  |
|  | 0.25 |  | 1997 |  |  |  |  |
|  | 0.2 |  | 1998 |  |  |  |  |
|  | 0.15 |  | 1999 |  |  |  |  |
|  | 0.1 |  | 2000 |  |  |  |  |
| Slovakia | 0.9 |  | 1988 | Nationwide |  | Stefancikova and Dubinsky 1995 | [68] |
|  | 0.9 |  | 1989 |  |  |  |  |
|  | 0.8 |  | 1990 |  |  |  |  |
|  | 0.7 |  | 1991 |  |  |  |  |
|  | 0.6 |  | 1992 |  |  |  |  |
|  | 0.5 |  | 1993 |  |  |  |  |
|  | 0.4 |  | 1994 |  |  |  |  |
| Slovakia | 0.25 |  | 1988 | Western Slovakia |  | Stefancikova and Dubinsky 1995 | [68] |
|  | 0.3 |  | 1989 |  |  |  |  |
|  | 0.2 |  | 1990 |  |  |  |  |
|  | 0.2 |  | 1991 |  |  |  |  |
|  | 0.15 |  | 1992 |  |  |  |  |
|  | 0.1 |  | 1993 |  |  |  |  |
|  | 0.04 |  | 1994 |  |  |  |  |
| Slovakia | 1.5 |  | 1988 | Central Slovakia |  | Stefancikova and Dubinsky 1995 | [68] |
|  | 1.7 |  | 1989 |  |  |  |  |
|  | 1.5 |  | 1990 |  |  |  |  |
|  | 1.5 |  | 1991 |  |  |  |  |
|  | 1.3 |  | 1992 |  |  |  |  |
|  | 0.79 |  | 1993 |  |  |  |  |
|  | 0.79 |  | 1994 |  |  |  |  |
| Slovakia | 1.5 |  | 1988 | Eastern Slovakia |  | Stefancikova and Dubinsky 1995 | [68] |
|  | 1.5 |  | 1989 |  |  |  |  |
|  | 1.2 |  | 1990 |  |  |  |  |
|  | 0.79 |  | 1991 |  |  |  |  |
|  | 0.79 |  | 1992 |  |  |  |  |
|  | 0.79 |  | 1993 |  |  |  |  |
|  | 0.63 |  | 1994 |  |  |  |  |
| Slovenia | 0.30 | 441/146968 | 1965-1991 | Abattoir level | Cattle slaughtered at Slaughterhouse "Pomurka" in Murska Sobota, Republic of Slovenia | Zivkovic et al., 1996 | [62] |
| Ukraine |  | 6 | 2005 |  | OIE database | <http://www.oie.int/wahis_2/public/wahid.php/Diseaseinformation/statusdetail>, Accessed 2017 |  |
| Ukraine | 0.024 | 5/20715 | 2012 | Abattoir level southern part of Ukraine | Publication | Khimich and Brodovsky, 2015 | [69] |
|  | 0.017 | 3/17181 | 2013 |  |  |  |  |
|  | 0.020 | 4/19242 | 2014 |  |  |  |  |

**References**

1. Fabijanić D, Giunio L, Ivaniš N, Fabijanić A, Mirić D, Kardum D. Ultrasonographic appearance of colon taeniasis. J Ultrasound Med. 2001;20:275–7.

2. Karanikas ID, Sakellaridis TE, Alexiou CP, Siaperas PA, Fotopoulos AC, Antsaklis GI. Taenia saginata: a rare cause of bowel obstruction. Trans R Soc Trop Med Hyg. 2007;101:527–8.

3. Lalošević, Vesna (Faculty of Agriculture NS (Serbia). D of VM, Kuruca, Ljiljana (Faculty of Agriculture NS (Serbia). D of VM, Simin, Stanislav (Faculty of Agriculture NS (Serbia). D of VM, Kozoderović, Gordana (Institute for Public Health of Vojvodina NS (Serbia)), Radosavljević, Biljana (Institute for Public Health of Vojvodina NS (Serbia)). Bovine cysticercosis in Serbia: is there a need for alternative diagnostic approach? Proc Int Conf Biol Food Saf Qual [Internet]. 2012. p. 128–30. Available from: http://www.vet.bg.ac.rs/~namirnice/download/SKUPOVI/BFSQ Belgrade 2012/Zbornik BFSQ/Zbornik radova BFSQ konferencija (1).pdf

4. Zammarchi L, Strohmeyer M, Bartalesi F, Bruno E, Muñoz J, Buonfrate D, et al. Epidemiology and Management of Cysticercosis and Taenia solium Taeniasis in Europe, Systematic Review 1990-2011. PLoS One. 2013;8.

5. Kurdova R. New trends in parasitism in Bulgaria [2]. Trends Parasitol. 2001. p. 314–5.

6. Lassen B, Janson M, Viltrop A, Neare K, Hütt P, Golovljova I, et al. Serological evidence of exposure to globally relevant zoonotic parasites in the Estonian population. PLoS One. 2016;11:164142.

7. Nasilowska M. [Cestode infections--1990]. Przegl Epidemiol. Poland; 1992;46:131–5.

8. Plonka W. [Taeniasis in 1992]. Przegl Epidemiol. Poland; 1994;48:149–54.

9. Plonka W. [Taeniasis in 1993]. Przegl Epidemiol. Poland; 1995;49:189–93.

10. Plonka W. [Taeniasis in 1995]. Przegl Epidemiol. Poland; 1997;51:161–5.

11. Plonka W. [Taenia infections in 1996]. Przegl Epidemiol. Poland; 1998;52:139–43.

12. Plonka W. [Cestode infections in 1997]. Przegl Epidemiol. Poland; 1999;53:159–65.

13. Plonka W. [Cestode infections in 1998]. Przegl Epidemiol. Poland; 2000;54:181–7.

14. Plonka W. [Cestode infections in Poland in 1999]. Przegl Epidemiol. Poland; 2001;55:159–63.

15. Plonka W, Waloch M. [Cestode infections in Poland in 2000]. Przegl Epidemiol. Poland; 2002;56:357–61.

16. Waloch M. [Cestode infections in Poland in 2001]. Przegl Epidemiol. Poland; 2003;57:159–63.

17. Waloch M. [Cestode infections in Poland in 2002]. Przegl Epidemiol. Poland; 2004;58:165–9.

18. Waloch M. [Cestode infections in Poland in 2003]. Przegl Epidemiol. Poland; 2005;59:331–5.

19. Waloch M. [Cestode infections in Poland in 2004]. Przegl Epidemiol. Poland; 2006;60:509–13.

20. Waloch M. [Cestode infections in Poland in 2005]. Przegl Epidemiol. Poland; 2007;61:305–9.

21. Waloch M. [Cestode infections in Poland in 2006]. Przegl Epidemiol. Poland; 2008;62:351–5.

22. Waloch M. [Cestode infections in Poland in 2007]. Przegl Epidemiol. Poland; 2009;63:267–9.

23. Waloch M. [Cestode infections in Poland in 2008]. Przegl Epidemiol. Poland; 2010;64:261–4.

24. Waloch M. [Cestode infections in Poland in 2009]. Przegl Epidemiol. Poland; 2011;65:285–8.

25. Economides P. Control of zoonoses in Cyprus. Rev Sci Tech. France; 2000;19:725–34.

26. Cuperlovic K. Current status of food-borne parasitic zoonoses--eastern Europe. Southeast Asian J. Trop. Med. Public Health. 1991. p. 72–7.

27. Derylo A, Szilman P. [Occurrence of human taeniasis and cysticercosis in pigs and cattle in the Katowice district]. Wiad Parazytol. Poland; 1995;41:443–54.

28. Rudohradská P, Halánová M, Ravaszová P, Goldová M, Valenčáková A, Halán M, et al. Prevalence of intestinal parasites in children from minority group with low hygienic standards in Slovakia. Helminthologia. 2012. p. 63–6.

29. Yong JL, Warren BA. Neurocysticercosis: a report of four cases. Pathology. England; 1994;26:244–9.

30. Davies MA, Turner J, Bentivoglio P. Spinal and basilar extraparenchymal neurocysticercosis. J Clin Neurosci Off J Neurosurg Soc Australas. Scotland; 1996;3:174–7.

31. Gubbay AD, Brophy BP, Henley S, Sage M. Neurocysticercosis. J Clin Neurosci Off J Neurosurg Soc Australas. Scotland; 1998;5:203–7.

32. Finsterer J, Kladosek A, Lubec D, Auer H. Bilateral thalamic stroke due to neurocysticercosis in a non-endemic area. Cerebrovasc. Dis. Switzerland; 2001. p. 354–6.

33. Talan-Hranilovic J, Sajko T, Negovetic L, Lupret V, Kalousek M. Cerebral cysticercosis and echinococcosis: a preoperative diagnostic dilemma. Arch Med Res. United States; 2002;33:590–4.

34. Titlic M, Tonkic A, Jukic I, Lahman-Doric M, Kolic K, Buca A, et al. Neurocysticercosis--non-specific clinical and neuroradiological presentation. Bratisl Lek Listy. Slovakia; 2007;108:414–6.

35. Vanista J, Lapkova E, Uhlikova M. [Cysticercosis in the Czech Republic]. Cesk Epidemiol Mikrobiol Imunol. Czech Republic; 1993;42:187–9.

36. Valkounova J, Zdarska Z, Slais J. Histochemistry of the racemose form of Cysticercus cellulosae. Folia Parasitol (Praha). Czech Republic; 1992;39:207–26.

37. Juhl ZK, Logager VB. [Subcutaneous cysticercosis and neurocysticercosis]. Ugeskr Laeger. Denmark; 2000;162:6691–2.

38. Palasis S, Drevelengas A. Extramedullary spinal cysticercosis. Eur J Radiol. Ireland; 1991;12:216–8.

39. Paterakis KN, Kapsalaki E, Hadjigeorgiou GM, Barbanis S, Fezoulidis I, Kourtopoulos H. Primary spinal intradural extramedullary cysticercosis. Surg Neurol. United States; 2007;68:309–11; discussion 312.

40. Meszaros I, Doczi T, Gomori E. [Current aspects in neurocysticercosis]. Orv Hetil. Hungary; 2003;144:1731–4.

41. Aksiks I, Sverzickis R. Neuronavigation guided surgery for parenchymal neurocysticercosis in two patients. Acta Neurochir (Wien). 2007;149:1169–72.

42. Vasiljevic-Vuckovic V, Milosevic Medenica S, Grujicic D. Neurocysticercosis mimicking brain tumor. Neuroradiol J. United States; 2011;24:419–23.

43. Soba B, Beovic B, Luznik Z, Skvarc M, Logar J. Evidence of human neurocysticercosis in Slovenia. Parasitology. England; 2014;141:547–53.

44. Bauer TM, Bruhwiler J, Aschwanden M, Wagner S, Schwander J. [Neurocysticercosis]. Dtsch Med Wochenschr. Germany; 1994;119:175–9.

45. Mestrovic T, Sviben M, Vilibic-Cavlek T, Ljubin-Sternak S, Tabain I, Mlinaric-Galinovic G. Seroprevalence of Taenia solium infections in Croatian patients presenting with epilepsy. J Helminthol. England; 2012;86:259–62.

46. Kolarova L. [Tissue helminthoses]. Klin. Mikrobiol. Infekc. Lek. Czech Republic; 2006. p. 131–4.

47. Czarkowski MP, Golab E. Invasive tapeworm infections in Poland in 2011. Przegl Epidemiol. Poland; 2013;67:263-266,365-367.

48. Golab E, Czarkowski MP. Echinococcosis and cysticercosis in Poland in 2012. Przegl Epidemiol. Poland; 2014;68:279-282,379-381.

49. Waloch M. [Cystic echinococcosis in Poland in 2010]. Przegl Epidemiol. Poland; 2012;66:311–3.

50. Doder R, Madle-Samardzija N, Canak G, Vukadinov J, Turkulov V, Sevic S. [Neurocysticercosis--5 years’ experience at the Clinic for Infectious Diseases]. Med Pregl. Serbia; 2002;55:523–7.

51. Nikolic A, Djurkovic-Djakovic O, Bobic B. [Intestinal parasitic infections in Serbia]. Srp Arh Celok Lek. Serbia; 1998;126:1–5.

52. Milovanović Aleksandar, Milovanović J., Gvozdenović Eleonora, Trivić A., Konstantinović Ljubica, Dulović Olga, Đukić Ana, Sanja K-T. Neurocysticercosis-work ability evaluation. Acta Vet Brno. 2008;58:139–47.

53. Devleesschauwer B, Allepuz A, Dermauw V, Johansen M V., Laranjo-González M, Smit GSA, et al. Taenia solium in Europe: Still endemic? Acta Trop. 2017;165:96–9.

54. Dorbek-Kolin E, Åhlberg T, Tummeleht L, Tappe D, Johansen MV, Lassen B. Prevalence of cysticercosis in Estonian pigs and cattle. Parasitol Res. 2018;117:591–5.

55. Dorny P, Vallée I, Alban L, Boes J, Boireau P, Boué F, et al. Development of harmonised schemes for the monitoring and reporting of Cysticercus in animals and foodstuffs in the European Union. EFSA Support Publ. Wiley Online Library; 2010;7:34E.

56. Lis H. Results of veterinary inspection of slaughtered animals and meat in Poland in 2000. Med Weter. POLISH SOCIETY OF VETERINARY SCIENCES; 2002;58:267–9.

57. Lis H. An evaluation of veterinary inspection of slaughtered animals and meat in Poland between 1987-1997. Med Weter. POLISH SOCIETY OF VETERINARY SCIENCES; 1999;55:243–6.

58. Lis H. Evaluation of the results of veterinary inspection of pigs slaughtered in Poland in 1994. Med Weter. POLSKIE TOWARZYSTWO NAUK WETERYNARYJNYCH AKADEMICKA 12, 20-033 LUBLIN, POLAND; 1998;54:417–20.

59. Kozlowska-Loj J. Prevalence of cysticercosis in cattle and pigs in the Lublin province in the years 2005-2008. Wiad Parazytol. Poland; 2011;57:193–4.

60. Kozlowska-Loj J, Loj-Maczulska A. Prevalence of cysticercosis in cattle and pigs in the Lublin province in the years 2009-2012. Ann Parasitol. Poland; 2014;60:309–10.

61. Oleleu AM, Gherman CM, Blaga R, Gy??rke A, Cozma V. Seroprevalence of porcine cysticercosis and influence of some associated risk factors in Northwestern Romania. Acta Vet Brno. 2016;85:121–6.

62. Živković J, Velimirović D, Džaja P, Grabarević Ž. Prevalenc of Cysticerus Bovis S Inermis Measles with Particular Reference to Histopathological Changes in Meat. Arch fur Leb Hyg. 1996;47:66.

63. Zdolec N, Vujević I, Dobranić V, Juras M, Grgurević N, Ardalić D, et al. Prevalence of Cysticercus bovis in slaughtered cattle determined by traditional meat inspection in Croatian abattoir from 2005 to 2010. Helminthologia. Springer; 2012;49:229–32.

64. Lis H. Evaluation of veterinary inspection of slaughtered cattle in Poland. Med Weter. POLSKIE TOWARZYSTWO NAUK WETERYNARYJNYCH AKADEMICKA 12, 20-033 LUBLIN, POLAND; 1997;53:155–8.

65. Oleleu AM, Gherman C, Cozma V. Prevalence of Taenia saginata larvae infection in cattle from Northwestern Romania (2009-2013). [Internet]. Sci. Parasitol. 2015. p. 47–52. Available from: http://www.scientia.zooparaz.net

66. Novakov N, Ćirković M, Aleksić N, Ljubojević D. Massive cysticercosis as a problem in cattle production in Serbia. Proc Int Conf Biol Food Saf Qual. Faculty of Agriculture, Novi Sad (Serbia); 2012.

67. Aleksic and Miloradovic. Cysticercosis in cattle slaughtered at the Pozarevac meat factory in Pozarevac, Serbia. Vet Glas. 1994;48:751–6.

68. Stefancikova A, Dubinsky P. Status and prognosis of the incidence of helminthic zoonoses in Slovakia. Helminthologia. VERSITA SOLIPSKA 14A-1, 02-482 WARSAW, POLAND; 1995;32:247–50.

69. Khimich MS, Brodovsky VA. ВЕТЕРИНАРНО-САНІТАРНА ОЦІНКА ЯЛОВИЧИХ ТУШ ПРИ УРАЖЕНІ ЦИСТИЦЕРКОЗОМ. Sci Messenger LNU Vet Med Biotechnol. 2015;17:426–30.

1. Age in years [↑](#footnote-ref-1)
2. Sol: abbreviated for *Taenia solium* [↑](#footnote-ref-2)
3. Sag: abbreviated for *Taenia saginata* [↑](#footnote-ref-3)
